# Use of a small molecule integrin activator as a systemically administered vaccine adjuvant in controlling Chagas disease

**DOI:** 10.1038/s41541-021-00378-5

**Published:** 2021-09-08

**Authors:** Nandadeva Lokugamage, Imran H. Chowdhury, Ronald J. Biediger, Robert V. Market, Sayadeth Khounlo, Navin D. Warier, Shen-An Hwang, Jeffrey K. Actor, Darren G. Woodside, Upendra Marathi, Peter Vanderslice, Nisha Jain Garg

**Affiliations:** 1 Department of Microbiology and Immunology, University of Texas Medical Branch (UTMB), Galveston, TX USA; 2 Department of Molecular Cardiology, Texas Heart Institute, Houston, TX USA; 3 Department of Pathology and Laboratory Medicine, UTHealth McGovern Medical School, Houston, TX USA; 4 7 Hills Pharma LLC, 2450 Holcombe Blvd, Suite J, Houston, TX USA; 5 Institute for Human Infections and Immunity, UTMB, Galveston, TX USA

**Keywords:** Diseases, Immunology

## Abstract

The development of suitable safe adjuvants to enhance appropriate antigen-driven immune responses remains a challenge. Here we describe the adjuvant properties of a small molecule activator of the integrins αLβ2 and α4β1, named 7HP349, which can be safely delivered systemically independent of antigen. 7HP349 directly activates integrin cell adhesion receptors crucial for the generation of an immune response. When delivered systemically in a model of Chagas disease following immunization with a DNA subunit vaccine encoding candidate *T. cruzi* antigens, TcG2 and TcG4, 7HP349 enhanced the vaccine efficacy in both prophylactic and therapeutic settings. In a prophylactic setting, mice immunized with 7HP349 adjuvanted vaccine exhibited significantly improved control of acute parasite burden in cardiac and skeletal muscle as compared to vaccination alone. When administered with vaccine therapeutically, parasite burden was again decreased, with the greatest adjuvant effect of 7HP349 being noted in skeletal muscle. In both settings, adjuvantation with 7HP349 was effective in decreasing pathological inflammatory infiltrate, improving the integrity of tissue, and controlling tissue fibrosis in the heart and skeletal muscle of acutely and chronically infected Chagas mice. The positive effects correlated with increased splenic frequencies of CD8^+^T effector cells and an increase in the production of IFN-γ and cytolytic molecules (perforin and granzyme) by the CD4^+^ and CD8^+^ effector and central memory subsets in response to challenge infection. This demonstrates that 7HP349 can serve as a systemically administered adjuvant to enhance T cell-mediated immune responses to vaccines. This approach could be applied to numerous vaccines with no reformulation of existing stockpiles.

## Introduction

Vaccines have proven to be one of the most effective treatments for preventing life-threatening infectious diseases. Despite this success, vaccines developed towards emerging diseases remain elusive due to inadequate induction of the immune response^1^. Although adjuvants are required to enhance the effectiveness of most vaccines, conventional adjuvants have limited effectiveness in high-risk subjects such as children and the elderly, and economic, safety and tolerability issues have hindered research into new adjuvants^2,3^. This is highlighted by the fact that the only FDA-approved adjuvant for many decades was aluminum salts (e.g., Alum)^1^. Clearly, a more diverse array of adjuvants is needed to accommodate the large variety of potential vaccine antigens^4^. For example, Alum fails to induce a robust Th1 response required for clearance of intracellular pathogens such as *Trypanosoma cruzi* (Chagas disease) and *Mycobacterium tuberculosis* (Tuberculosis)^1^. Historically, the effectiveness of adjuvants has been somewhat empirically determined. More recently approved adjuvant components including monophosphoryl lipid A and cytosine phosphoguanine and other agents still under evaluation are believed to bind pattern recognition receptors, e.g., Toll-like receptors, to trigger an innate immune response^1^. As the immune response has become better understood, approaches to finely target specific receptors and mediators to enhance antigen presentation, T cell co-stimulation, and Th1 vs. Th2 cytokines production, are being evaluated^4^.

Integrin cell adhesion molecules are abundantly expressed on lymphocytes and play an essential role in shaping the immune response^5^. Although identified as key regulators of lymphocyte homing and recirculation, they are now recognized as critical regulators of T cell activation and differentiation during antigen presentation and effector functions^6–9^. Integrins αLβ2 (also called lymphocyte function-associated molecule-1, LFA-1) and α4β1 (also called very late activation antigen-4, VLA-4) were among the initial costimulatory receptors identified^6–9^. Prolonged integrin-mediated interactions between T cells and antigen-presenting cells (APC), particularly via the αLβ2/ICAM-1 axis, are required for effective long-term T cell-mediated memory^10^. In addition, αLβ2 and α4β1 are clinically validated drug targets; their inhibition was effective in dampening the over-active immune responses causing psoriasis^11^, dry-eye disease^12^, multiple sclerosis^13^, and inflammatory bowel disease^14^. The immunosuppressive nature of integrin antagonist therapy is underscored by the observation of reactivation of latent viral infections such as multifocal leukoencephalopathy (caused by JC virus) when targeting either integrin αLβ2^15^ or α4β1^16^. Based on the clinical observations that inhibition of αLβ2 and α4β1 is immunosuppressive, we hypothesized that activation of these integrins would be immunostimulatory and may prove useful as adjuvants to further vaccine development.

We have previously described and extensively characterized an initial lead compound, THI0019, as a small molecule agonist of α4β1 integrin^17^. THI0019 significantly enhances cell adhesion to VCAM-1 under both static and flow conditions, as well as facilitates α4β1-dependent cell rolling, spreading, and migration^17^. THI0019, as well as close structural analogs, were also found to facilitate αLβ2 integrin/ICAM-1 induced adhesion and migration^17^, identifying it as a small molecule activator of both αLβ2 and α4β1 integrins. Here, we describe an analog of THI0019, 7HP349, and tested whether systemic administration of 7HP349 would enhance the prophylactic and therapeutic efficacy of a candidate DNA vaccine against Chagas disease.

Chagas Disease, caused by *Trypanosoma cruzi (T. cruzi or Tc)*, is a major health concern in Latin America and it is an emerging disease in the United States, Europe, Japan, and other countries^18^. Most individuals exposed to *Tc* remain seropositive for life. In ~30% of infected persons, clinical symptoms progress from cardiac hypertrophic remodeling to dilated cardiomyopathy and ultimately result in cardiac arrest and death^19^. To evaluate the effectiveness of 7HP349 as a systemic adjuvant to potentially improve the immunity to intracellular infectious pathogens, we chose a murine model of *T. cruzi* infection because (1) a T cell-mediated immune response is required for the control of this pathogen, (2) the vaccines are currently an active area of development (including the use of recombinant subunit DNA vaccines) for Chagas disease, and (3) there is a compelling clinical unmet need for such vaccines in both prophylactic and therapeutic settings.

## Results

### 7HP349 enhances α4β1-mediated and αLβ2-mediated cell adhesion in vitro and functions as an adjuvant in vivo

**Fig. 1:**
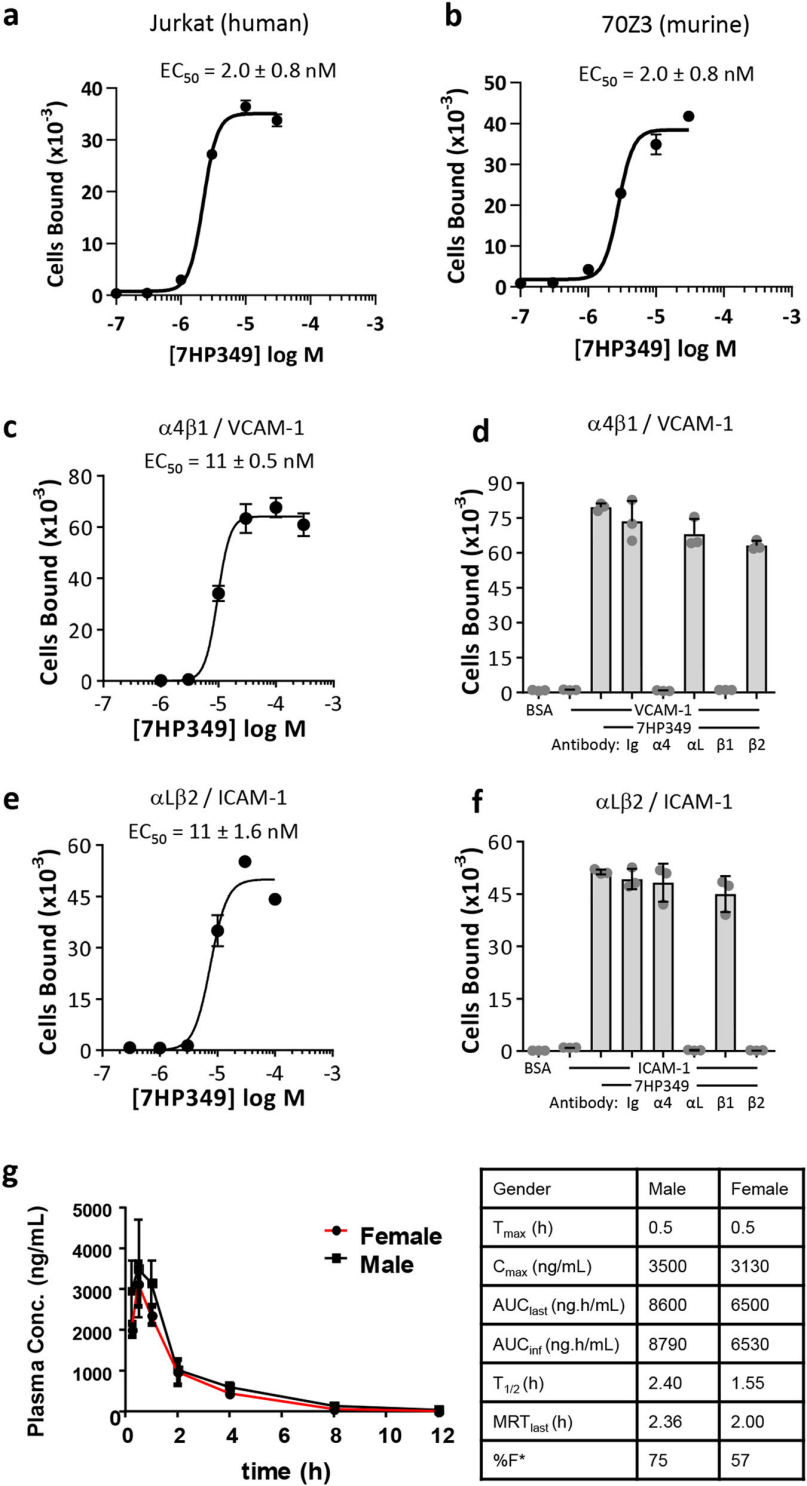
Activity and bioavailability of integrin activator 7HP349. **a** Dose-response curves of 7HP349 against human (**a**) and mouse (**b**) α4β1. The compound activates cell adhesion to VCAM-1 (**c**, **d**) and ICAM-1 (**e**, **f**) in an α4β1 and αLβ2 dependent fashion, respectively. All adhesion assays were performed at least three times, and data are presented as mean ± SD from triplicate determinations. One representative experiment is shown. Mean EC_50_ values determined from 3 independent experiments are shown either in the absence (**a**, **b**) or presence (**c**, **e**) of 50% serum. All function-blocking mAbs and IgG controls were used at 10 µg/mL. **g** Male and female C57BL/6 mice were dosed intraperitoneally (40 mg/kg), and blood samples were collected at the indicated time points (*n* = 3 mice/time point). The plasma samples were analyzed for the quantification of 7HP349 using a fit-for-purpose LC-MS/MS method and PK parameters calculated using non-compartmental analysis. Data are plotted as mean values ± standard error mean (SEM).

Our initial integrin activator, THI0019, has been extensively characterized^17^. It markedly enhances binding of both α4β1 and αLβ2 to their cognate integrin ligands, vascular cell adhesion molecule 1 (VCAM-1) and intercellular adhesion molecule 1 (ICAM-1), respectively, with EC_50_ values in the 1–2 µM range, and enhances cell adhesion under both static and flow conditions. To determine whether a structural analog of THI0019 could enhance integrin-dependent cell adhesion, we tested 7HP349 in standard α4β1-dependent and αLβ2-dependent adhesion assays. Like THI0019, 7HP349 enhances the binding of the human T cell line Jurkat to VCAM-1 with an EC_50_ of 2 µM (Fig. 1a). The 7HP349 has virtually identical activity against the mouse α4β1 receptor, evidenced by enhanced binding of the murine B-cell line 70Z/3 to VCAM-1 (Fig. 1b). In both species, the number of cells bound to VCAM-1 increased by >100-fold in the presence of 7HP349. When static cell adhesion assays were performed under more physiological conditions in buffers containing 50% serum, the EC_50_ curves shifted to the right suggesting the compound is likely to be moderately protein-bound in vivo (Fig. 1c). The activity against α4β1 is completely blocked by antibodies to either the α4 or β1 subunits of the integrin (Fig1d). In a similar fashion, 7HP349 enhanced cell adhesion to ICAM-1 in an αLβ2-dependent fashion (Fig. 1e, f).

Given the role played by αLβ2 and α4β1 integrins in the generation of an immune response^7^, we were interested in evaluating the potential of 7HP349 to serve as an adjuvant for vaccines. For this purpose, we determined the pharmacokinetics (PK) of 7HP349 in mice. Following a single IV bolus administration of 7HP349 (4-mg/kg), the compound was determined to have moderate plasma clearance and a somewhat elevated volume of distribution. Terminal plasma half-life was found to be ~2 h. 7HP349 (40 mg/Kg) was formulated in PBS/glycerol/tween-80 vehicle to be used for either admix or separate systemic delivery. Intraperitoneal administration of the compound resulted in reasonable exposure levels (57–75% of IV exposure) with marginally higher exposures in males than females based on *C*_max_ and AUC_last_ values (Fig. 1g). Half-lives were consistent with that determined for IV administration.

**Fig. 2:**
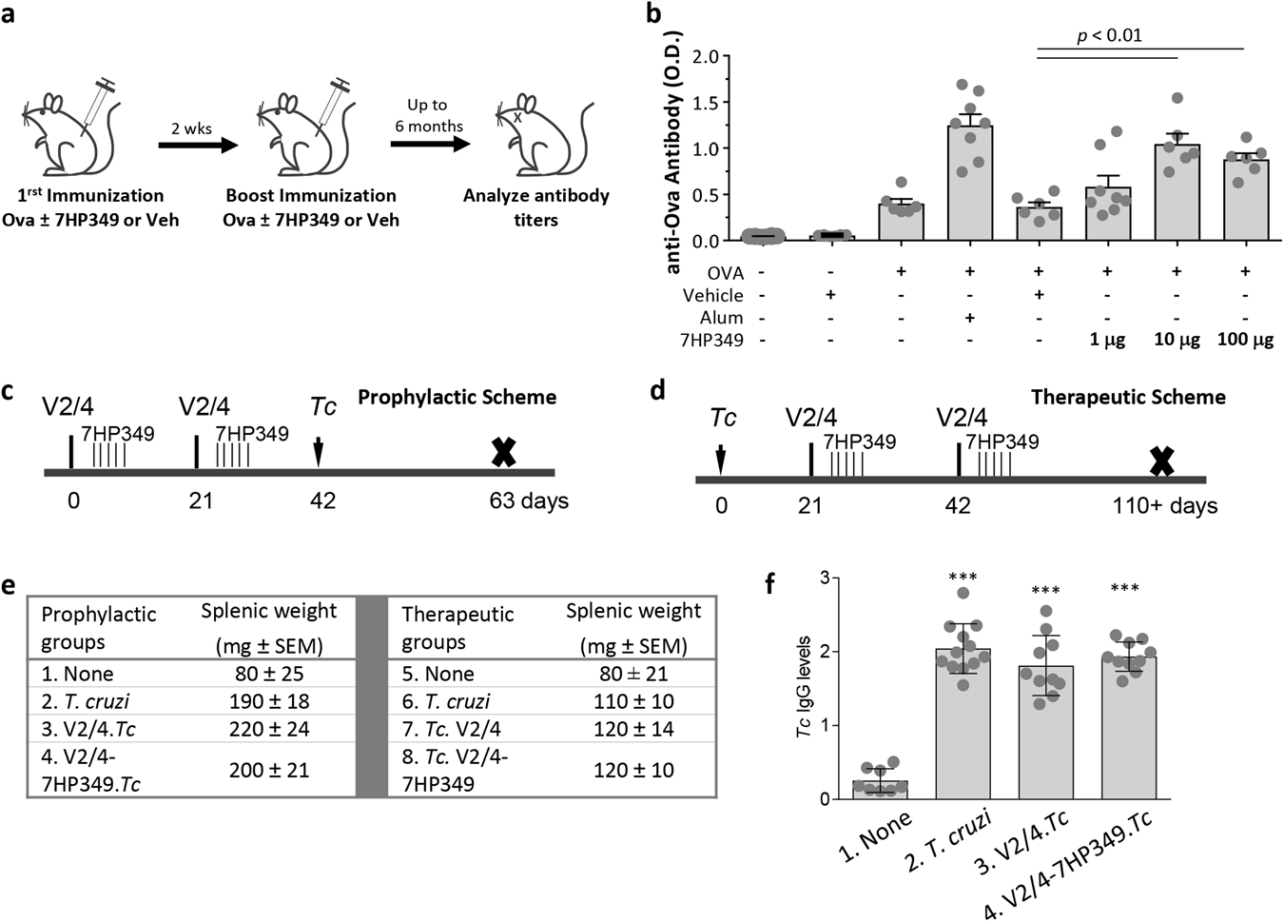
Adjuvantation of humoral responses with 7HP349. **a** Ovalbumin (OVA) immunization and boost scheme. **b** Comparative OD values representing antibody titers generated at 2 months post-immunization and boost using OVA adjuvanted with 7HP349. C57BL/6 mice were immunized and boosted at 14 days. Sera were serially diluted and tested for antibody reactivity against OVA by ELISA. Relative OD values (450 nm) for each group are graphed at dilutions of 1:25,600. Mean values ± SEM are plotted (*n* = 6–8 values per group). P values were determined by 1-way analysis of variance (ANOVA) with Tukey’s post-hoc test (comparison of multiple groups). **c**, **d** Chagas disease and immunization models. Mice (C57BL/6, females, 6 weeks age) were immunized with two doses of TcG2/TcG4-encoding DNA vaccine (V2/4) at 21-day intervals. 7HP349 was inoculated in 5 daily doses, beginning 1 day after DNA vaccine delivery. Challenge infection was carried out by intraperitoneal injection of 10,000 parasites per mouse. **c** Mice given the prophylactic vaccine were euthanized 21 days later corresponding to the acute infection phase. **d** Mice given the therapeutic vaccine were euthanized at 110 days post-infection corresponding to the chronic disease phase. **e** Splenic wet weight (mean value in mg ± SEM). **f** Serum levels of anti-*T. cruzi* antibodies in prophylactically immunized/infected mice were monitored by an ELISA. Data are presented as mean ± SD and significance (non-infected vs. infected or vaccinated/infected) is annotated as ***p < 0.001. *P* values in bar graphs (**b**, **f**) were determined by 1-way analysis of variance (ANOVA) with Tukey’s post-hoc test (comparison of multiple groups).

Prior to testing in a more complex model and dosing regimen, we performed a simple pilot study in a mouse ovalbumin challenge model to determine if 7HP349 had any adjuvant-like activity. In this design, 7HP349 was admixed with the ovalbumin similar to most classic adjuvants. C57BL/6 mice were immunized with ovalbumin admixed with vehicle or 7HP349 (1, 10, or 100 μg) (Fig. 2a). Mice receiving 7HP349 or vehicle alone, or ovalbumin in alum served as negative and positive controls, respectively. Figure 2b summarizes titer results obtained 2-months post-boost immunization. Titers from mice dosed with ovalbumin in combination with alum or 7HP349 (10 or 100 µg) were significantly increased above titers from mice given ovalbumin alone or ovalbumin mixed with the vehicle. There was no statistical difference in titers between the alum-treated and 7HP349-treated (10 or 100 µg) groups. Together, these results indicate that 7HP349 adjuvants the αLβ2 and α4β1 dependent adhesion of immune cells and activation of humoral immunity like that noted with Alum. Alum is, however, not known to induce a significantly robust T cell response typically needed to clear intracellular pathogens^1^ such as *T. cruzi*, the ultimate target of a Chagas disease vaccine. We further characterized the 7HP349 adjuvating properties in enhancing the T cell response in a Chagas disease model.

### Chagas vaccine model

Previously, we have shown that C57BL/6 wild‐type mice infected with 10,000 *T. cruzi* trypomastigotes (SylvioX10 isolate) exhibit parasitemic phase for 7–40 days post-infection (pi). Pathologic changes associated with inflammatory infiltrate, oxidative stress, and fibrosis can be observed after 90 days pi, and left ventricular dysfunction is noted at ~6-months pi^20^. We employed this well‐established experimental model to examine the role of 7HP349 in improving the protection offered by a 2-component DNA vaccine (referred to as V2/4). Prophylactic efficacy was evaluated against the acute parasitemic phase (Fig. 2c), and therapeutic efficacy was evaluated against the chronic phase of parasite persistence and tissue pathology (Fig. 2d). Since the vaccine is DNA-based, 7HP349 was not premixed with the vaccine, but rather dosed intraperitoneally 1-day post-vaccination and then daily for a total of 5 days. Our intent was to provide coverage over the typical time course of initiation of T cell activation.

Acutely infected mice (±prophylactic vaccine) exhibited a 137.5–175% increase in the expansion of splenic cells (V2/4.*Tc* > V2/4-7HP349.*Tc* > *Tc* only; spleen weight: 190–220 mg in groups 2–4 vs. 80 mg in controls, Fig. 2e). Chronically infected mice (±therapeutic vaccine) exhibited a 37.5–50% increase in splenic cells (*Tc*.V2/4-7HP349 = *Tc*.V2/4 > *Tc* only; spleen weight: 110–120 mg in groups 6–8 vs. 80 mg in controls, Fig. 2e). All mice, irrespective of vaccination regimen, responded to *T. cruzi* infection with a significant increase in serum levels of anti-parasite antibodies (Fig. 2f). No adverse reactions were observed in mice injected with DNA vaccine with or without 7HP349 adjuvant. These findings indicate that exposure to *T. cruzi* results in potent expansion of splenic cells and antibody response and 7HP349 is safe to deliver with a DNA vaccine. Considering DNA vaccine primarily elicits T cell response^21^, it was not surprising to observe no significant effects of the vaccine (±7HP349) on the parasite-specific antibody levels in infected mice.

### 7HP349 effects on vaccine-induced T cells phenotypic profile in *T. cruzi* infected mice

CD4^+^ and CD8^+^T cells play a key role in the immune control of intracellular pathogens. To determine if and how 7HP349 modulates the V2/4-induced T cell phenotypic profile in *T. cruzi* infection, mice were vaccinated, challenged, and euthanized at 21 days pi (as in Fig. 2c). Splenocytes were either analyzed immediately without in vitro stimulation or incubated in vitro with *Tc* antigenic lysate to examine the recall response. The representative flow cytometry gating strategy to capture CD3^+^T cell populations is shown in Supplementary Fig. 1a–d. We cumulatively analyzed the concatenated fcs files from no treatment/no infection, non-vaccinated/infected, V2/4.*Tc*, and V2/4-7HP349.*Tc* groups by FlowSOM (1 × 10^5^ of CD3^+^ live cells per mouse, *n* = 5–10 per group) that created self-organizing 14 meta-clusters (P0-P13). The tSNE plots of the T cell metaclusters from all groups of mice before and after in vitro stimulation with antigenic lysate are shown in Supplementary Fig. 1k–r. The unbiased phenotypic designation of the metaclusters (Supplementary Fig. 1s) was based on the differences in the expression levels of CD8, CD4, CD25, CD62L, and CD44, presented in the heatmap in Supplementary Fig. 1t. This analysis identified CD4^−^CD8^−^ (double negative, DN), CD4^+^ and CD8^+^T cell clusters that were further distinguished into naïve (TN, CD44^−^CD62L^+^), effector/effector memory (TEM, CD44^+^CD62L^−^), and central memory (TCM, CD44^hi^CD62L^+^) phenotypes. The intermediate (int) states of the naive (TN^int^, low median fluorescence intensity (MFI) of CD62L than in TN state), TEM (TEM^int^, low CD44 MFI than in TEM), and TCM (TCM^int^, high CD44 MFI than in TCM) cells was captured by variability in the expression levels of CD44 and CD62L molecules. Mean values ± standard error mean (SEM) and statistical significance for the subpopulations in each group are presented in Supplementary Table 1.

**Fig. 3:**
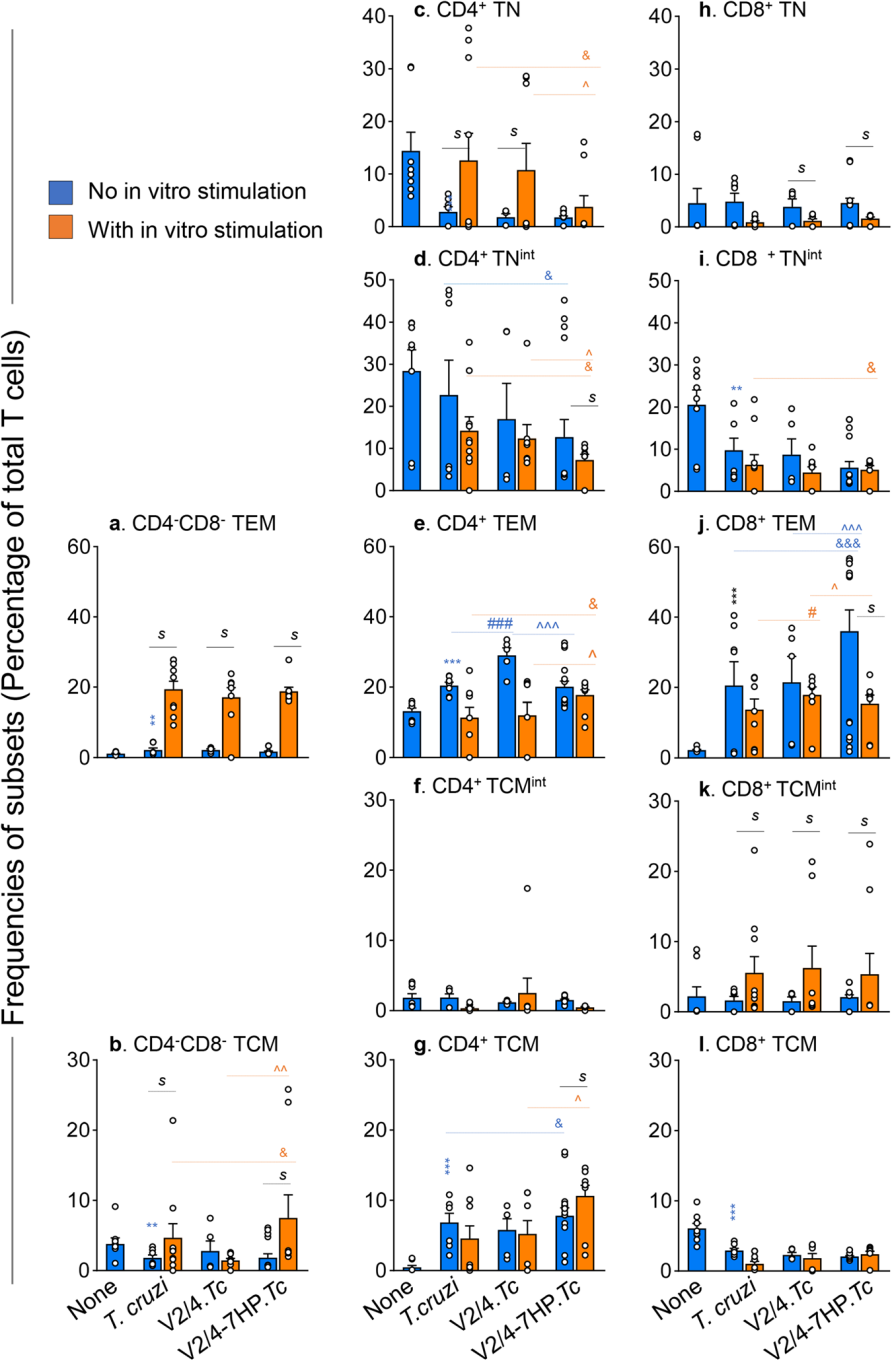
Effects of 7HP349 adjuvant on vaccine-induced T cells phenotype in *T. cruzi* infection. C57BL/6 female mice were prophylactically immunized with V2/4 in the presence or absence of 7H349, challenged with *T. cruzi*, and euthanized at 21 days post-infection as is described in Fig.2c. Splenocytes were either used immediately (blue bars) or incubated for 48 h with *T. cruzi* antigenic lysate (orange bars). Cells were labeled with fluorescent-conjugated antibodies and analyzed by flow cytometry. Self-organizing meta-clusters of CD3^+^T cells based on the expression levels of CD4, CD8, CD62L, and CD44 antigens were formed by FlowSOM analysis. The bar graph shows percent positive of CD4^−^CD8^−^ (**a**, **b**), CD4^+^ (**c**–**g**), and CD8^+^ (**h**–**l**) T cell subsets that exhibited naïve (TN, **c**, **h**), naïve intermediate (TN^int^, **d**, **i**), effector/effector memory (TEM, **a**, **e**, **j**), central memory intermediate (TCM^int^, **f**, **k**) and central memory (TCM, **b**, **g**, **i**) phenotype in no treatment/no infection, non-vaccinated/infected, V2/4.*Tc* and V2/4-7HP349.*Tc* groups of mice. Data are plotted as mean values ± SEM (*n* = 5–10 mice per group). Significance between control vs. infected mice (*) was calculated by unpaired *t*-test or Mann-Whitney U test. Significance among infected groups was calculated by 1-way analysis of variance (ANOVA) with Tukey’s post-hoc test (comparison of multiple groups) and plotted as ^#^infected vs. V2/4.*Tc*, ^infected vs. V2/4-7HP349.*Tc*, and ^&^V2/4-7HP349 vs. V2/4. *P* values of <0.05, <0.01, and <0.001 are annotated with one, two, and three symbols, respectively. The horizontal bar indicates the compared groups. In some graphs, the horizontal bar with *s* indicates significance (*p* < 0.05) between matched groups before and after in vitro stimulation.

Ex vivo analysis (i.e., without in vitro stimulation) showed that the DN TEM, DN TCM, CD4^+^ premature, and CD4^+^ TCM^int^ subsets constituted a small proportion (i.e., <3%) of total T cells in all infected and control groups of mice (Fig. 3a, b, f, Supplementary Table 1, blue bars**)**. All infected mice exhibited a general decline in the splenic percentages of CD4^+^ TN and CD4^+^ TN^int^ subsets (Fig. 3c, d, blue bars) and an increase in the CD4^+^ TEM and CD4^+^ TCM subsets (Fig. 3e, g, blue bars). Moreover, frequencies of CD8^+^ TN and CD8^+^ TCM^int^ were not changed after infection (Fig. 3h, k, blue bars), CD8^+^ TN^int^ and CD8^+^ TCM subpopulations were decreased in all infected (vs. control) groups (Fig. 3i, l, blue bars**)**, while CD8^+^ TEM subset (8.2–15.1-fold) expanded in infected mice (Fig. 3j, blue bars). Notably, maximal expansion of the CD8^+^ TEM subset was observed in V2/4-7HP349.*Tc* group (Fig. 3j, blue bars).

The T cells profile after in vitro stimulation with *Tc* antigenic lysate is shown in orange bars in Fig. 3. The splenic DN TEM and DN TCM subsets of infected mice were expanded upon 2nd antigenic stimulation, and maximal increase in DN TCM subset was noted in V2/4-7HP349.*Tc* group (Fig. 3a, b, compare blue and orange bars). The CD4^+^ TN subset was increased by >3-fold, CD4^+^ TN^int^ and CD4^+^ TEM subsets were shrunk by 33–58.5%, and the CD4^+^ TCM subset exhibited non-significant changes in response to in vitro antigenic exposure in non-vaccinated/infected and V2/4.*Tc* mice (Fig. 3c–g, compare blue and orange bars). In comparison, stable frequencies of CD4^+^ TN and CD4^+^ TEM subsets and an increase in the CD4^+^ TCM subset were noted in V2/4-7HP349.*Tc* mice after in vitro antigenic stimulation (Fig. 3c, e, g, compare blue and orange bars). Regarding CD8^+^T cells, all infected mice exhibited a general decline in CD8^+^ TN, CD8^+^ TN^int^, and CD8^+^ TEM subsets after in vitro antigenic exposure (Fig. 3h–j) that indicates that CD8^+^T cells likely apoptosed after primary activation. More of the CD8^+^T cells exhibited TCM^int^ (1.5–3.1-fold increase) phenotype and CD8^+^ TCM cells were maintained after in vitro stimulation in all infected groups (Fig. 3k, l). These results suggest that (a) 7HP349 adjuvant maximally enhanced the splenic frequencies of CD8^+^ TEM subset while decreasing the naïve T cells in infected mice. Further, (b) 7HP349 adjuvanted (vs. non-adjuvanted) vaccine imparted maximal expansion of the splenic DN TCM and CD4^+^ TCM subsets in response to 2^nd^ antigenic stimulation. Overall, 7HP349 adjuvant enhanced the activation of the effector/effector memory phenotype of the CD4^+^ and CD8^+^T cells in infected mice.

### 7HP349 effects on functional activation of vaccine-induced T cells in *T. cruzi* infected mice

**Fig. 4:**
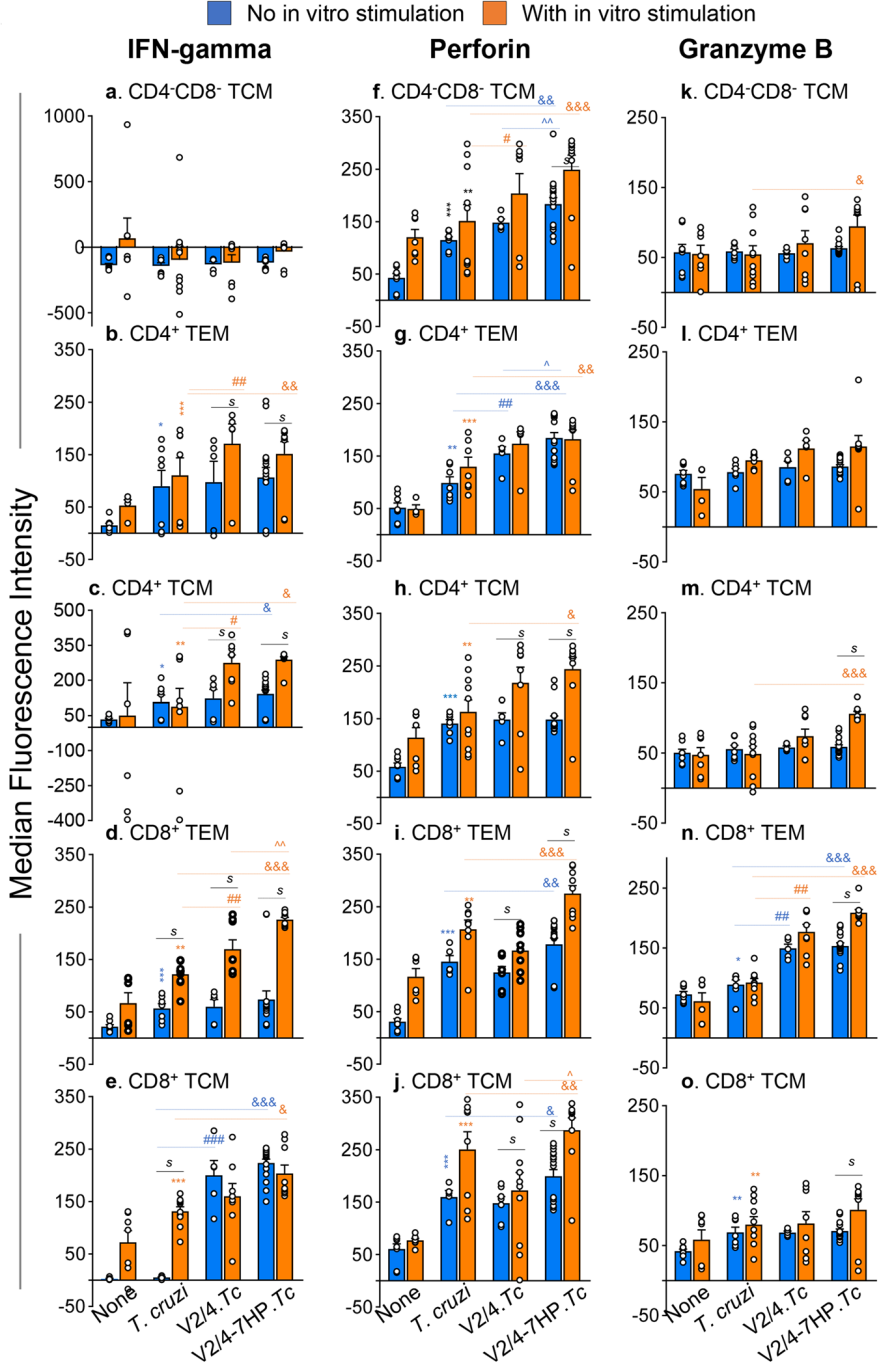
Effects of 7HP349 adjuvant on vaccine-induced T cells function in *T. cruzi* infection. C57BL/6 female mice were immunized, challenged, and euthanized as in Fig. 2c. Splenocytes were either used immediately (blue bars) or incubated for 48 h with *T. cruzi* antigenic lysate (orange bars). Cells were then labeled with fluorescence-conjugated antibodies and analyzed by flow cytometry. The median fluorescent intensities for IFN-γ (**a**–**e**), perforin (**f**–**j**), and granzyme B (**k**–**o**) in CD4^−^CD8^−^, CD4^+^, and CD8^+^T cell subsets with effector/effector memory (TEM) and T central memory (TCM) phenotype are plotted. Data are plotted as mean values ± SEM (*n* = 5–10 mice per group). Significance between control vs. infected groups (*) was calculated by unpaired *t*-test or Mann–Whitney U test. Significance among infected groups was calculated by 1-way analysis of variance (ANOVA) with Tukey’s post-hoc test (comparison of multiple groups) and plotted as ^#^infected vs. V2/4.*Tc*, ^infected vs. V2/4-7HP349.*Tc*, and ^&^V2/4-7HP349 vs. V2/4. *P* values of <0.05, <0.01, and <0.001 are annotated with one, two, and three symbols, respectively. The horizontal bar indicates the compared groups. In some graphs, the horizontal bar with *s* indicates significance (*p* < 0.05) between matched groups before and after in vitro stimulation.

Functional analysis of T cell subsets in infected and vaccinated mice (±7HP349) before and after in vitro stimulation is shown in Fig. 4 and Supplementary Table 2. IFN-γ production by DN TCM was negligible (Fig. 4a). Ex vivo analysis showed that IFN-γ production by CD4^+^ TEM, CD4^+^ TCM, and CD8^+^ TEM subsets was increased by 1.59–6.19-fold in infected (vs. control) groups, and the maximal increase was noted in V2/4-7HP349.*Tc* mice (Fig. 4b–d, Supplementary Table 2, blue bars). A potent increase in IFN-γ production by CD8^+^ TCM subset (67–75.9-fold) was noted in vaccinated mice only (V2/4-7HP349.*Tc* > V2/4.*Tc*, Fig. 4e, blue bars). Upon in vitro stimulation, IFN-γ production by CD4^+^ TEM, CD4^+^ TCM, and CD8^+^ TEM subsets was further expanded by 1.41-2.06-fold in vaccinated/infected mice (V2/4-7HP349.*Tc* *>* V2/4.*Tc*), while CD8^+^ TCM remained stable in vaccinated/infected mice (Fig. 4b–e, compare blue and orange bars). Non-vaccinated/infected mice exhibited an increase in IFN-γ production in the CD8^+^ TCM subset only after in vitro antigenic stimulation only (Fig. 4e).

Regarding the expression of cytolytic molecules required for intracellular control of the pathogen, we noted a 0.91–4.77-fold increase in perforin (PFN) expression in the majority of T cell subsets in infected groups (V2/4-7HP349.*Tc* > V2/4.*Tc* > *Tc*, Fig. 4f–j, blue bars). PFN production by these T cell subsets was further expanded by 30.55–64.39% after in vitro stimulation in vaccinated/infected mice only (V2/4-7HP349.*Tc* > V2/4.*Tc*, Fig. 4f–j, compare blue and orange bars). Granzyme B (GZB) expression in T cell subsets was not generally changed in infected (vs. control) mice except that GZB production by CD8^+^ TEM subset was increased by 105–111% in vaccinated mice (Fig. 4n, blue bars). In response to in vitro antigenic stimulation, a 33–80% increase in GZB production by all T cell subsets was observed in V2/4-7HP349.*Tc* mice only (Fig. 4k–o, compare blue and orange bars).

**Fig. 5:**
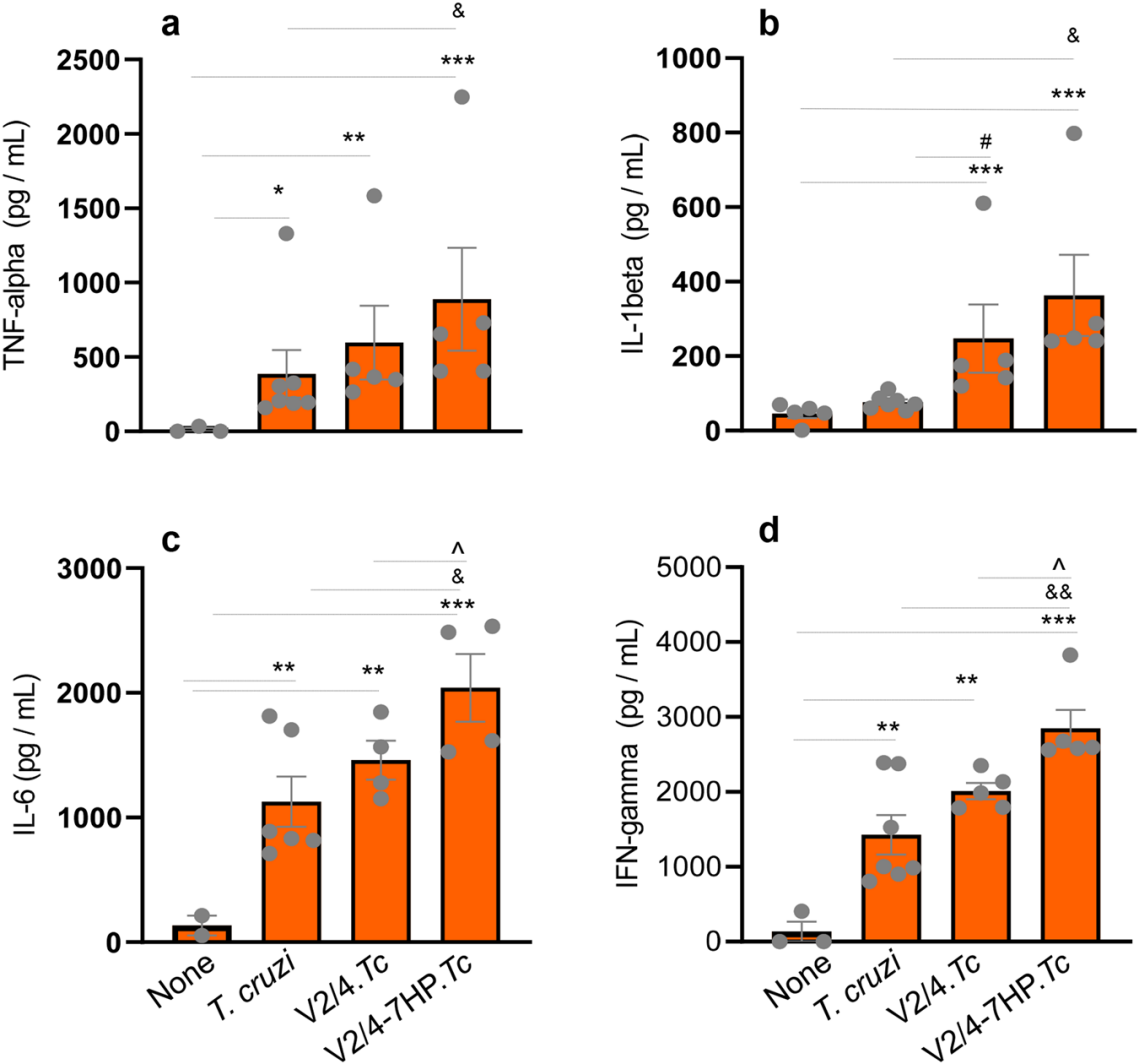
Cytokine release by splenocytes of *T. cruzi* infected mice immunized with the vaccine (±7HP349). C57BL/6 female mice were immunized, challenged, and euthanized as in Fig.2c. Splenocytes were incubated for 48 h with *T. cruzi* antigenic lysate. An ELISA was performed to measure the TNF-α (**a**), IL-1β (**b**), IL-6 (**c**), and IFN-γ (**d**) cytokines release in the supernatants. Data are derived from *n* = 5–10 mice per group (at least duplicate observations per sample) and plotted as mean values ± SEM. Significance between control vs. infected mice (*) was calculated by unpaired *t*-test or Mann–Whitney U test. Significance among infected groups was calculated by 1-way analysis of variance (ANOVA) with Tukey’s post-hoc test (comparison of multiple groups) and plotted as ^#^infected vs. V2/4.*Tc*, ^infected vs. V2/4-7HP349.*Tc*, and ^&^V2/4-7HP349 vs. V2/4. P values of <0.05, <0.01, and <0.001 are annotated with one, two, and three symbols, respectively. The horizontal bar indicates the compared groups.

An ELISA evaluation of the supernatants for cytokines released by splenocytes in vitro stimulated with *Tc* antigenic lysate is shown in Fig. 5. These results showed a significant increase in splenocytes’ release of TNF-α (Fig. 5a), IL-6 (Fig. 5c), and IFN-γ (Fig. 5d) in nonvaccinated/infected (vs. non-infected/control) mice (all, *p* < 0.05). Non-significant changes in IL-1β release were noted in infected (vs. control) mice (Fig. 5b). The splenocytes’ release of TNF-α and IL-1β was increased by 69% and 224.8%, respectively (all, ^&^*p* < 0.05) while IL-6 and IFN-γ release was not significantly changed when mice were prophylactically immunized with V2/4 vaccine before infection (vs. non-vaccinated/infected mice) (Fig. 5a–d). Importantly, splenocytes of mice in V2/4-7HP349.*Tc* group exhibited 167%, 378%, 102%, and 36.5% increase in TNF-α, IL-1β, IL-6, and IFN-γ release, respectively, as compared to that noted in splenocytes of non-vaccinated/infected mice (^&^*p* < 0.05–0.01, Fig. 5a–d).

Together, the results presented in Fig. 4 and Fig. 5, along with those discussed in Fig. 3, show that 7HP349 enhanced the T cell response in mice. Specifically, 7HP349 adjuvanted V2/4 vaccine led to maximal expansion and functional activation of CD4^+^ TCM and CD8^+^ TEM subsets and maintained stable production of cytokines and cytotoxic molecules by the CD4^+^ TEM subset in infected mice. Overall inflammatory cytokine response was maximally activated in mice given 7HP349 adjuvanted V2/4 vaccine.

### Control of tissue parasites

**Fig. 6:**
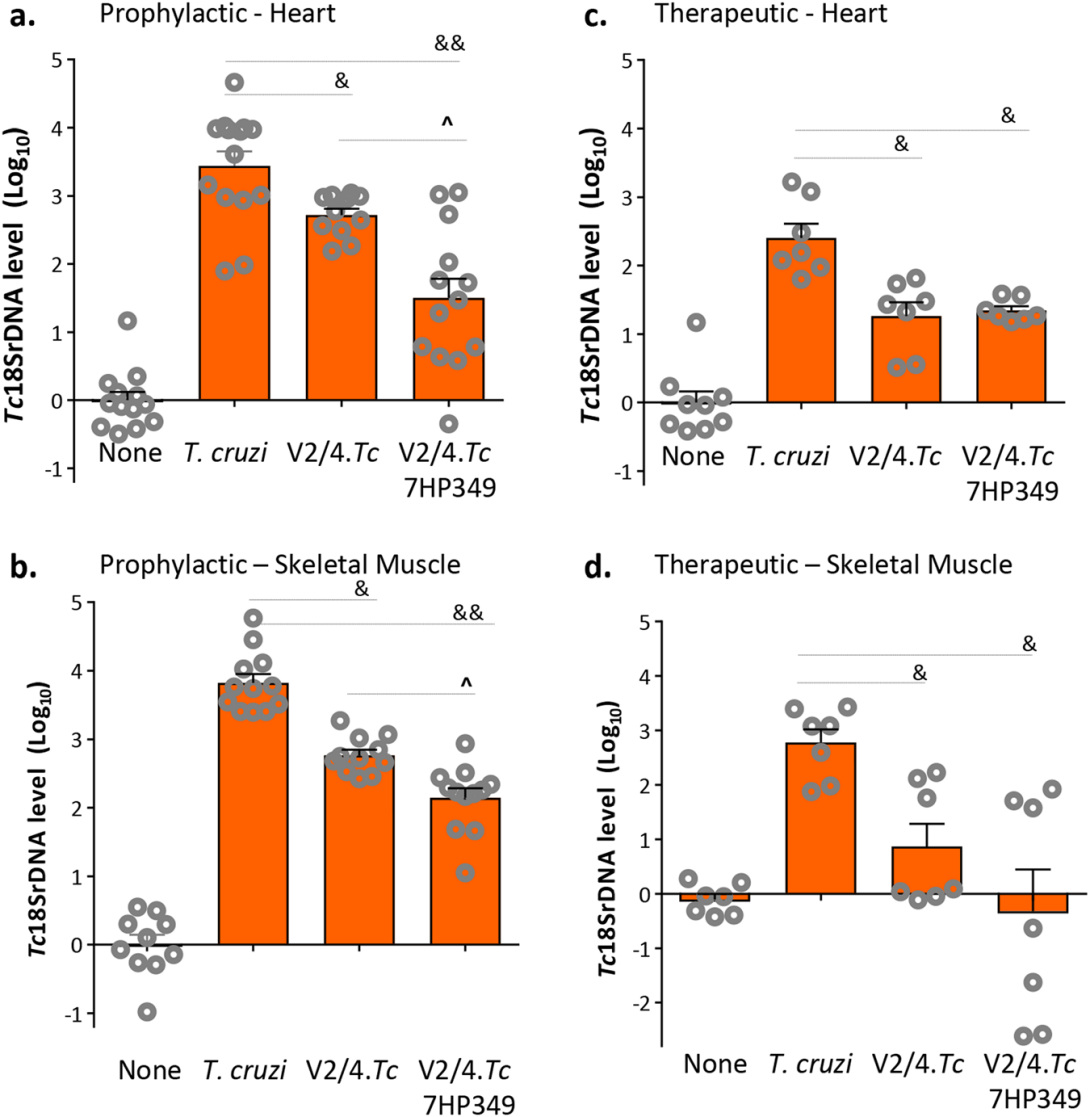
7HP349 improves the vaccine efficacy in controlling tissue parasite burden in Chagas mice. **a**, **b** Mice were given prophylactic vaccine with or without 7HP349 adjuvant, challenged, and euthanized at 21 days post-infection as in Fig. 2c. **c**, **d** For therapeutic studies, mice were challenged, vaccinated (±7HP349) at day 21 and 42 post-infection, and euthanized at 110^+^ days post-infection, as in Fig. 2d. Total DNA was isolated from the heart (**a**, **c**) and skeletal muscle (**b, d**) tissues and submitted to real-time qPCR amplification of *Tc*18SrDNA sequence (normalized to murine *GAPDH*). Data (mean ± SEM) are representative of duplicate observations per sample (*n * = 7–12 mice per group). Significance between control vs. infected groups (*) was calculated by unpaired *t*-test or Mann–Whitney U test. Significance among infected groups was calculated by 1-way analysis of variance (ANOVA) with Tukey’s post-hoc test (comparison of multiple groups) and plotted as ^#^infected vs. V2/4.*Tc*, ^infected vs. V2/4-7HP349.*Tc*, and ^&^V2/4-7HP349 vs. V2/4. *P* values of <0.05, <0.01, and <0.001 are annotated with one, two, and three symbols, respectively. The horizontal bar indicates the compared groups.

Next, we determined if 7HP349 modulation of immune responses enhanced the hosts’ ability to control tissue parasite burden. Mice challenged with *Tc* (without vaccination) exhibited very high levels of *Tc*18SrDNA in the myocardial and skeletal muscle tissues obtained during the acute infection phase (Fig. 6a, b) and persistence of parasites during the chronic phase (Fig. 6c, d). Prophylactic immunization with V2/4 resulted in 91% and 89.7% control of acute parasite burden in heart and skeletal muscle tissues (^&^*p* < 0.01) that was further decreased by 59.5% and 78.1%, respectively, by 7HP349 adjuvantation of the vaccine (^^^*p* < 0.05) (Fig. 6a, b). When V2/4 was given as a therapeutic vaccine, we noted 94.3% and 95.6% control of parasite persistence in cardiac tissue and skeletal muscle of immunized mice, respectively (Fig. 6c, d, ^&^*p* < 0.05). Mice given 7HP349 adjuvanted V2/4 as a therapeutic treatment exhibited no detectable parasite persistence in skeletal muscle tissue (Fig. 6d, ^*p* < 0.05). These results suggest that 7HP349 further improved the vaccine efficacy in reducing the acute and chronic tissue parasite burden and in providing protection against *Tc* infection in mice.

### Control of tissue inflammatory infiltrate and fibrosis by 7HP349 adjuvanted vaccine

**Fig. 7:**
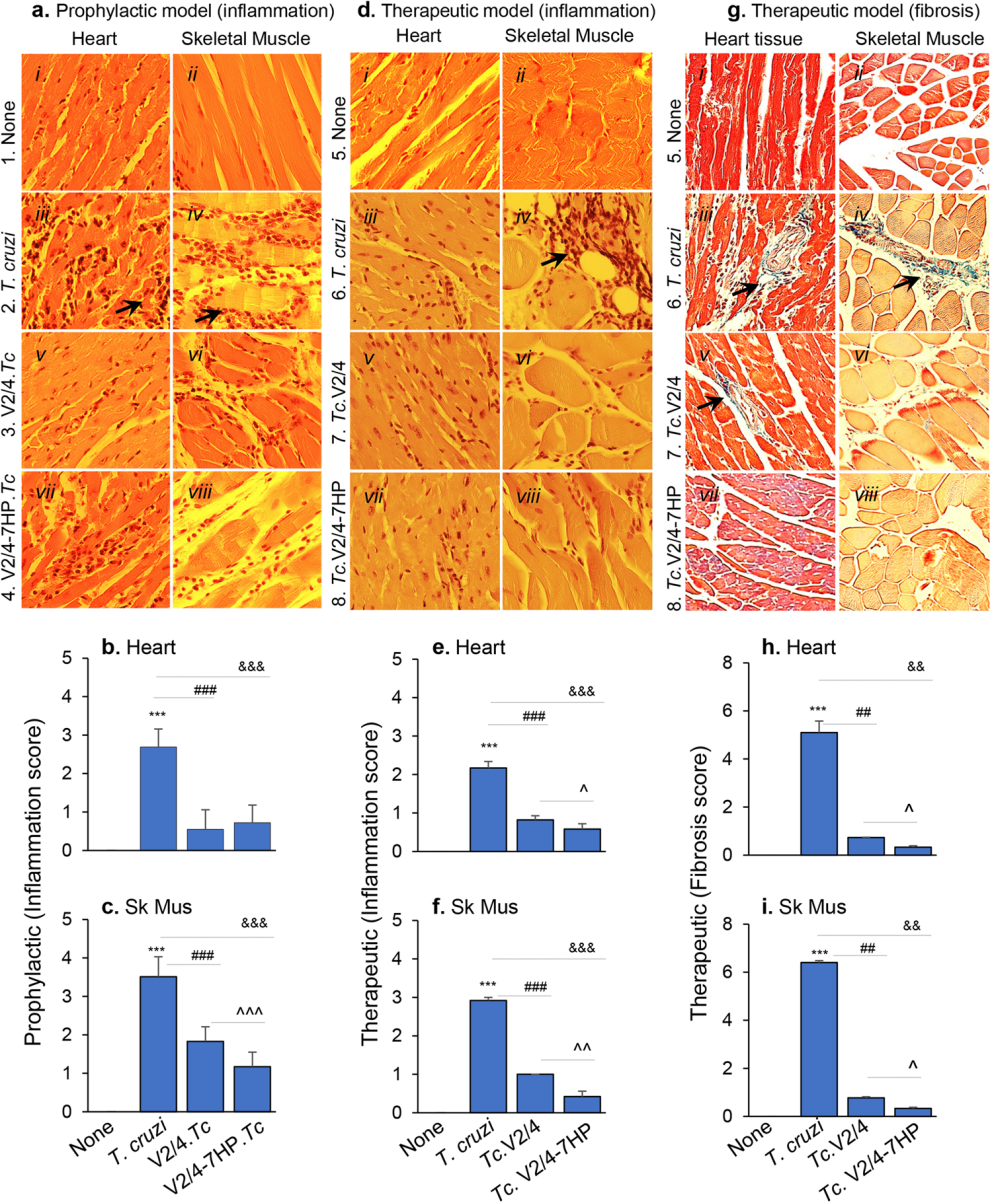
7HP349 adjuvants the prophylactic and therapeutic efficacy of the DNA vaccine in controlling tissue inflammatory infiltrate and cardiac fibrosis in Chagas mice. Mice were treated prophylactically (**a**–**c**) or therapeutically (**d**–**i**) as described in Fig. 2c, d. **a**–**f** Paraffin-embedded 5 µm heart and skeletal muscle tissue sections were examined by hematoxylin/eosin (H&E) staining (magnification: ×20). Representative H & E images of the heart and skeletal tissue sections from all groups are presented in **a** and **d** (panels i–viii). The inflammatory scores (mean values ± SEM derived from *n* = 3 mice per group, 2 tissue sections per mouse, >9 microscopic fields per tissue) are presented in **b**, **c**, **e**, **f**. **g**–**i** Paraffin-embedded 5 µm heart and skeletal muscle tissue sections were examined by Masson’s trichrome staining (collagen fibers: blue, nuclei: black, background: red; magnification: ×20). Representative Mason’s Trichrome images of the heart and skeletal tissue sections from all groups are presented in **g** (panels i-viii). The fibrosis scores (mean values ± SEM derived from *n* = 3 mice per group, 2 tissue sections per mouse, >9 microscopic fields per tissue) are presented in **h**, **i**. Significance between control vs. infected mice (*) was calculated by unpaired *t*-test or Mann–Whitney U test. Significance among infected groups was calculated by 1-way analysis of variance (ANOVA) with Tukey’s post-hoc test (comparison of multiple groups) and plotted as ^#^infected vs. V2/4.*Tc*, ^infected vs. V2/4-7HP349.*Tc*, and ^&^V2/4-7HP349 vs. V2/4. *P* values of <0.05, <0.01, and <0.001 are annotated with one, two, and three symbols, respectively. The horizontal bar indicates the compared groups.

We performed H&E and Mason Trichrome staining of tissue sections to assess the extent of tissue damage in Chagas mice (Fig. 7, Supplementary Table 3). Non-vaccinated, acutely infected mice exhibited extensive infiltration of inflammatory cells, disseminated inflammation, and loss of tissue integrity in their heart (histology score: 2–3) and skeletal muscle (histology score: 3–4) tissues when compared to non-infected mice (Fig. 7a*.* i–iv, b, c). Prophylactic immunization with V2/4 resulted in up to 80 and 48% decline in the inflammatory infiltrate in cardiac and skeletal muscle tissues, respectively (^#^*p* < 0.001), and adjuvantation with 7HP349 led to 36% (^*p* < 0.001) further decline in the inflammatory infiltrate in skeletal muscle of immunized mice (Fig. 7a*.* v–viii, b, c). Therapeutic vaccination also showed promising results in chronic CD mice (Fig. 7d–f). Pronounced infiltrate with diffused and focused inflammatory foci was noted in the heart (score: 0–3) and skeletal muscle (score: 1–3) tissue, respectively, of chronically infected (vs. non-infected) mice (Fig. 7d*.* i–iv, e, f). Skeletal tissue degeneration was particularly evident in non-treated CD mice (Fig. 7d*.* iv). Therapeutic delivery of V2/4 resulted in 62% and 65.8% decline in myocardial and skeletal muscle levels of inflammatory infiltrate, respectively, in chronically infected mice (Fig. 7d. v–vi, ^#^*p* < 0.001). Importantly, adjuvantation of V2/4 with 7HP349 led to a further 30% (^*p* < 0.05) and 58% (^*p* < 0.01) decline in myocardial and skeletal muscle levels of inflammatory pathology in chronically infected mice (Fig. 7d*.* vii–viii, e, f).

Masson’s-Trichrome staining of tissues revealed that 7HP349 adjuvant also enhanced vaccine efficacy in controlling the tissue fibrosis in Chagas mice (Fig. 7g–i). Noticeably large, blue-stained areas, indicative of infiltration of fibroblasts/myofibroblasts, and tissue scarring were evident in the heart (fibrosis score: 5.1) and skeletal muscle (fibrosis score: 6.4) of chronically infected (vs. non-infected, control) mice (Fig. 7g*.* i–iv, h, i). Therapeutic immunization of infected mice with V2/4 led to an 85–87% decline in myocardial and skeletal tissue levels of fibrotic response (^#^*p* < 0.01, Fig. 7g*.* v–vi, h, i). Importantly, adjuvantation of V2/4 with 7HP349 led to a further 54.8–57% (^*p* < 0.05) decline in myocardial and skeletal muscle levels of fibrotic response in chronically infected mice (Fig. 7g. vii–viii, h, i).

Together, the results presented in Fig. 7 suggest that 7HP349 enhanced the prophylactic and therapeutic efficacy of the DNA vaccine in controlling the tissue inflammatory infiltrate and improving the integrity of tissue in the heart and skeletal muscle of acutely and chronically infected Chagas mice. Such a decline in tissue inflammation by V2/4 and 7HP349 adjuvanted vaccine in acutely and chronically infected mice is highly impressive considering that the splenic generation of protective type 1 T cell immunity was enhanced by vaccination. Further, 7HP349 enhanced the therapeutic efficacy of the vaccine in eliminating tissue fibrosis in chronic Chagas mice.

## Discussion

In this study, we evaluated the potential of a small molecule activator of the integrins α4β1 and αLβ2 to serve as a vaccine adjuvant, specifically when administered systemically separate from the vaccine antigen. Using a candidate DNA-prime/DNA-boost vaccine, we demonstrate that dosing the compound after the vaccine enhances the protective efficacy against *T. cruzi* infection and persistence and Chagas disease pathology.

Subunit vaccines are the safest form of vaccine for infectious pathogens. In the context of Chagas disease, several candidate antigens have been tested as subunit vaccines, delivered in the form of DNA, recombinant protein, or a host of viral and bacterial expression vectors, in small animal models (reviewed in^22–24^). We developed and employed a computational/bioinformatic algorithm to screen the *Tc* sequence database and identify antigens that have characteristics of vaccine candidates^25^. Of the several candidate antigens selected, three candidates (TcG1, TcG2, and TcG4) passed several biological screens; these were highly conserved in clinically relevant *Tc* isolates, *e*xpressed (mRNA/protein) in mammalian forms (trypomastigote and amastigote) of *Tc*, and recognized by IgGs and/or type 1 CD8^+^T cell in infected mice^26^, dogs^27^, and humans^28^. We, therefore, have tested the vaccine potential of these three candidates using diverse prime-boost approaches in eliciting resistance to *Tc* infection. The homologous DNA-prime/DNA-boost approach was simplest in design and was tested in several compositions (individually or co-delivery of antigenic candidates, different doses, and time-intervals, with or without cytokine adjuvants etc.)^25,26,29^. The TcG1 candidate was least immunogenic; it stimulated antibody and T cell responses only when it was delivered with IL-12 and GM-CSF adjuvants, and, therefore, removed from the later formulations. Thus, we included TcG2 and TcG4 only in this study.

Proliferation and activation of antigen-specific cytotoxic T lymphocytes are essential for the control of pathogens like *Tc* that replicate in a variety of host cells. Integrins provide mechanical support and transduce signals to modulate T cell differentiation, motility, proliferation, and activation^30^. Mature T cells shuttle between peripheral organs and immune tissues. In this context, αLβ2 and α4β1 play a pivotal role, interacting with endothelial ICAM-1 and VCAM-1, in regulating T cell diapedesis, migration, and homing to target tissues^30^. αLβ2, by directly engaging the ICAM family of molecules on antigen-presenting cells (APCs), is one of the best-characterized costimulatory molecules in antigen recognition by T cell receptors and shaping the adaptive immune response to infectious agents^8,9^. The αLβ2/ICAM-1 axis is necessary for prolonged T cell and dendritic cell engagement needed to establish effective CD8^+^T cell memory^10^. Thus, we included 7HP349, a small molecule allosteric activator of integrin cell adhesion molecules αLβ2 and α4β1, as an adjuvant to enhance vaccine-induced T cell immunity against *Tc* infection.

Quality and quantity of T cell response, and their cytokine polyfunctionality are critical factors in defining the protective efficacy of vaccine(s) against intracellular pathogens. Immunodominant peptides binding stably to MHC class II on the surface of APCs enhance the recruitment and proliferation of effector CD4^+^T cells, and secretion of IFN-γ/TNF-α and chemokines by activated APCs and CD4^+^T cells enhances the CD8^+^ T cell response. IFN-γ and TNF-α play a major role in parasite clearance^31,32^ and the production of these cytokines together leads to greater killing than either cytokine alone. In addition, CD8^+^T (and to some degree CD4^+^T) cells mediate cytolytic activity through the release of perforin and granzymes^32^. Our data in this study show that adjuvantation with 7HP349 enhanced the T cell expansion and functional activation in mice. Specifically, 7HP349 adjuvanted vaccine the maximal expansion and functional activation of CD4^+^ TCM and CD8^+^ TEM subsets and maintained stable production of cytokines and cytotoxic molecules by CD4^+^ TEM subset in infected mice. Further, the 7HP349 adjuvanted vaccine enhanced the ability of infected mice to respond to re-exposure to pathogen antigens with increased frequencies of CD4^+^ and CD8^+^ effector T cells and increased production of IFN-γ and cytotoxic molecules by CD8^+^ effector and central memory cells and other type I cytokines by splenic cells. It is important to note that though systemic T cell response was increased, pathological inflammatory infiltrates and consequent fibrosis in tissues were reduced in vaccinated mice, thus, suggesting that DNA vaccine and 7HP349 did not cause non-specific tissue injury.

In conjunction with the enhanced T cell response, V2/4-7HP349 exhibited maximal efficacy in controlling the *Tc* infection. This was evidenced by our findings that V2/4 controlled ~90% of acute parasite burden, and 7HP349 adjuvantation further decreased the parasite burden by >60% in immunized mice. Likewise, therapeutic treatment with V2/4 resulted in 94–95% control of chronic parasite persistence, and 7HP349 adjuvantation led to complete clearance of parasite burden in the skeletal muscle of chronically infected mice. These findings support the idea that a vaccine designed to enhance systemic Th1, cytotoxic CD8^+^ T cell response is the best choice for preventing and/or controlling *Tc* infection. To the best of our knowledge, this is the best efficacy of V2/4 experimental vaccine we have achieved in a prophylactic setting and the best efficacy of any experimental vaccine in a therapeutic setting.

The persistence of inflammatory infiltrate associated with extensive fibrosis and necrosis was noted in the heart and skeletal muscle tissues of Chagas mice. With control of parasite persistence, we detected very low levels of tissue inflammatory infiltrate and improved tissue integrity in vaccine-treated/infected mice and better preservation of tissues was observed in mice treated with the 7HP349 adjuvanted vaccine. Gene expression profiling studies showed that upregulation of IFN-γ-inducible genes and elevated levels of TNFα were associated with worsened cardiac function in Chagas patients^33–35^. These and other studies conclude that while pro-inflammatory cytokine secreting CD4^+^T and CD8^+^T cells and cytotoxic T lymphocytes are essential for protection from infection, the persistence of these cells is detrimental and associated with tissue pathology and tissue damage in Chagas disease. Indeed, IL-10^+^T cells found predominantly in clinically asymptomatic Chagas patients^36^ play a vital role in the regulation of pathogenic responses. Our findings in this study provide compelling evidence that 7HP349 induced proliferation and activation of CD8^+^ T cells was parasite-specific, and after achieving parasite control, 7HP349 did not continue to stimulate non-specific pathological/inflammatory T cells in Chagas disease.

Along with the extensive research efforts dedicated to defining the appropriate antigens for the development of effective subunit vaccine against Chagas disease, it is also important that adjuvants that can adequately enhance the protective immune response elicited by a given vaccine are also identified and developed further. Some adjuvants have been evaluated in animal models using TcG2/TcG4 subunit vaccine with limited success^37,38^. *Trypanosoma rangeli* (*T. rangeli*) proteome shares significant homology with that of *T. cruzi* and has been shown when administered as an immunogen to protect mice against *T. cruzi* infection^39,40^. When glutaraldehyde fixed *T. rangeli* lysate was used as an adjuvant with the TcG2/TcG4 subunit vaccine in a DNA prime/DNA boost mouse model like that described here, no enhancement of vaccine efficacy against acute *T. cruzi* infection was detected^37^. In similar models using a TcG2/TcG4 DNA prime/protein boost vaccine, no adjuvantation was seen when IL-12 and GM-CSF expression plasmids were co-administered at the time of the DNA priming dose^38^.

In recent studies involving other vaccine prototypes, the synthetic TLR4 agonist E6020 has been used as an adjuvant in a squalene-based oil: water emulsion with the recombinant *T. cruzi* flagellar protein Tc24 and has proved effective in both acute and chronic settings^41,42^. Caeiro et al showed prophylactic protection using surface antigen TcTASV-C in combination with U-OMP19, given in an alum-saponin formulation^43^. Sanchez-Alberti et al have demonstrated that adjuvantation of a trivalent immunogen with a cyclic dinucleotide (CDN) proved effective in a mouse model of Chagas disease^44^. These studies support the idea that triggering a Th1-dependent cell-mediated immunity, along with a balanced Th17 response, is important for an adjuvant for Chagas vaccine preparations. The CDN is a known agonist of the STING receptor that recognizes cytosolic nucleic acid ligands and has been shown to activate the innate immune system (IRF3, NF-κB, STAT6). In contrast, the integrin activator described here, 7HP349, is an adjuvant that impacts the adaptive immune response via antigen presentation, immune cell trafficking, and killing response, independent of the TLR4 and STING mediated pathways^45^. In addition, E6020, U-OMP19, and CDN were admixed with the respective engineered antigen complexes prior to administration. One of the unique features of 7HP349 is that it can be dosed systemically in a manner independent of the vaccine antigen. This suggests applicability to numerous other vaccines, whether they be subunit or nucleic acid-based, without requiring reformulation of existing stockpiles. 7HP349 has been shown to be orally available in rats and dogs and thus an oral formulation may be an easy means of adjuvanting an existing vaccine (unpublished observations). Unformulated CDNs alone cause significant inflammation and necrosis in tissues with high doses of systemic administration^44,46^. 7HP349 alone shows no reactivity or toxicity and is highly safe to deliver. Interestingly, E6020 alone shows some levels of protection against Chagas disease as a systemically administered agent, although it does not reduce cardiac inflammation and fibrosis^47^. 7HP349 was not tested as a single agent in this study. Pivotal Investigational New Drug (IND)-enabling rat and dog safety and toxicity studies have been completed and have demonstrated a large safety margin and therapeutic window for 7HP349 upon systemic administration. As a result, the FDA has recently activated an IND application with Phase 1 human studies initiated in October 2020^48^. As a systemic drug, 7HP349 has the potential to not only augment recruitment of APCs to the site of the vaccine but also enable prolonged ICAM-1-αLβ2 integrin-mediated adhesion of APCs and naïve T cells in lymph nodes known to be important for memory^10^. In doing so, 7HP349 may be useful in augmenting prophylactic responses to newly designed vaccines, as well as improve adjuvantation of current vaccines such as influenza in the elderly, where age-related defects in dendritic cells’ ICAM-1 induction have been observed^49^.

In summary, this study demonstrates that systemic activation of integrins may safely augment the efficacy of existing and new vaccines. The small molecule 7HP349 adjuvant enhances a Chagas vaccine-stimulated pro-inflammatory cytokine production and cytotoxic activity of CD4^+^ and CD8^+^ TEM cells and provides better control of acute parasitemia, as well as parasite persistence. The mechanism of action of 7HP349 through activation of the integrins αLβ2 and α4β1 suggests it could be effective in combination with a variety of antigens and thus may represent a universal adjuvant for several vaccines. Furthermore, since it is dosed systemically independent of the antigen, it may represent an effective adjunct component that does not require the reformulation of existing vaccine preparations. Further studies examining 7HP349 side by side with other adjuvants in modulating the host response to infectious agents (virus, bacteria, parasites) in multiple animal models are warranted.

## Materials and methods

### Ethics statement

All animal experiments were conducted following the National Institutes of Health guidelines for housing and care of laboratory animals and in accordance with protocols approved by the Institutional Animal Care and Use Committees at The University of Texas Medical Branch at Galveston (protocol number 08-05-029) and the UTHealth McGovern Medical School. All experiments were conducted in ABSL2/BSL2-approved laboratory and all personnel has received appropriate ABSL2/BSL2 training. All authors complied with the institutional and NIH guidelines and ethical regulations for animal research.

### Reagents and cell lines

The complete chemical synthesis of integrin activator 7HP349 is included in the patent^50^. For all in vitro assays, compounds were dissolved in DMSO to make a series of stock solutions such that a 1:100 dilution in assay buffer or media would yield the desired final working concentrations in 1% DMSO (vehicle). For in vivo studies, 7HP349 was formulated in a vehicle made from individual components on a weight/weight basis. The vehicle is a PBS solution, pH 7.4, containing 17% Tween 80, 16% glycerol. 7HP349 was prepared at 0.02, 0.2, and 2 mg/g (Ovalbumin study) or 0.5 mg/g (Chagas study). Vehicle and 7HP349 solutions were filtered through a 0.22 µm filter and pipetted into sterile septum vials. The final compound concentration (mg/mL) was verified by HPLC compared to a standard curve. Human VCAM-1 and ICAM-1 were purchased from R&D Systems (Minneapolis, MN). Antibodies were purchased from ABD Serotec (Raleigh, NC) (HP2/1 [anti-α4] and 38 [anti-αL]). The mAb 33B6 [anti-β1] was a gift from B. McIntyre (MD Anderson Cancer Center, Houston, TX). The cell lines Jurkat, HSB, and 70Z/3 were obtained from American Type Culture Collection (Manassas, VA) and were maintained in recommended culture media.

### Static cell adhesion assays

Ligands (VCAM-1, ICAM-1) in 50 µL of 50 mM Tris-HCl (pH 7.4), 150 mM NaCl (TBS) were added to wells of a 96-well plate and allowed to coat overnight at 4 °C. To maximize the window to evaluate agonist activity, a sub-optimal coating concentration of ligand was used. This ligand concentration corresponded approximately to that which would yield ≤5% adhesion as determined by dose-response curves of ligand binding to the appropriate cell type. Unless otherwise stated in figure legends, the concentrations of ligands used for the assays were 0.5 µg/mL VCAM-1 and 5 µg/mL ICAM-1^51^. Briefly, 2 × 10^6^ cells were labeled for 30 min with calcein-AM (Molecular Probes), washed, resuspended in binding buffer, and added to triplicate wells of ligand-coated plates (2 × 10^5^ cells/well) that had been blocked with 2% BSA. After a 30-min incubation at 37 °C, the plates were washed 3 times with binding buffer, the adherent cells were lysed, and fluorescence was measured on a Tecan Safire^2^ plate reader. Standard curves were run for each assay to convert fluorescence units to cell numbers. For each assay, the cells expressed the appropriate integrin receptor endogenously (human Jurkat/α4β1, HSB/αLβ2). The binding buffers were PBS with 1 mM MgCl_2_ for α4β1 and TBS with 2 mM MgCl_2_, 5 mM EGTA for αLβ2 assays. For assays in the presence of serum, 50% FBS was included in the buffers. Mouse (70Z/3) assays were performed in TBS with 1 mM MnCl_2_. Human or mouse VCAM-1 concentrations for the assays without serum were 1 µg/mL and 0.5 µg/mL, respectively. When used, mAbs at 10 µg/mL were premixed with the cells prior to adding to the wells. All assays were performed 3 times and data are presented as mean EC_50_ ± standard deviation.

### Pharmacokinetic studies

C57BL/6 mice were administered a single dose of 7HP349 either IV (4 mg/Kg in 5% (v/v) DMSO + 10% (v/v) Cremophor EL + 35% (v/v) PEG400 + 0.1 M Citrate buffer pH: 2.7) or IP (40 mg/Kg in PBS, pH 7.4, containing 17% tween 80, 16% glycerol). Blood samples were collected (*n* = 3 mice/time point) at 0.25, 0.5, 1, 2, 4, 8, 12, and 24 h post-dose. At each time point, about 120 µL of blood was collected by retro-orbital sinus puncture into a labeled microfuge tube containing 200 mM K_2_EDTA solution (20 µL per mL of blood). All plasma samples were stored below −60 °C until bioanalysis. The plasma samples were analyzed for the quantification of 7HP349 using a fit-for-purpose LC-MS/MS method with a lower limit of quantification (LLOQ) of 7.50 ng/mL. The pharmacokinetic parameters of 7HP349 were calculated using the non-compartmental analysis tool of validated Phoenix^®^ WinNonlin^®^ software (version 6.3).

### Ovalbumin (OVA) model

OVA in PBS was mixed 1:1 with 7HP349 stock solutions (0.02, 0.2, and 2 mg/mL) to yield mixtures containing 50 µg OVA with 1, 10, or 100 µg 7HP349 in 100 µL. Mixtures were injected subcutaneously at the base of the tail into 6-week-old female C57BL/6 mice. Control mice received OVA mixed with alum (aluminum salt, aluminum hydroxide). Negative controls included naïve mice (no treatment) and mice treated with just OVA (no adjuvant), OVA mixed with the vehicle, or just vehicle alone (no antigen). Mice were boosted at 14 days post initial immunization and then bled by retro-orbital sinus puncture at 1-month, 2-months, 4-months, and 6-months following boost. The schematic view of OVA immunization is presented in Fig. 2a. Sera samples were serially diluted and tested for antibody reactivity against OVA using an ELISA.

### Chagas model: vaccine composition, immunization, and challenge infection

The cDNAs for TcG2 and TcG4 (Genbank: AY727915 and AY727917, respectively) were cloned in pCDNA3.1 eukaryotic expression plasmid^25^. Recombinant plasmids were transformed into *E*. *coli* DH5-alpha-competent cells, and purified by anion exchange chromatography by using a Qiagen Endofree maxi prep kit (Qiagen, Chatsworth, CA)^25^. Purified plasmids were used at a concentration of 25 μg each plasmid per vaccination. When used, 7HP349 was delivered at 1 mg/Kg once daily for 5 days.

C57BL/6 female mice (6-week-old) were obtained from Jackson Laboratory (ME, USA). *T*. *cruzi* (*Tc*) trypomastigotes (Sylvio X10/4 strain, ATCC 50823) were maintained and propagated by continuous in vitro passage in C2C12 cells^52^ and used for infection of mice (10,000 parasites/mouse, intraperitoneal). Each dose of DNA vaccine (referred to as V2/4) was constituted of 25-µg of each plasmid (*pCDNA3.TcG2* and *pCDNA3.TcG4*) and delivered in 50 µL PBS by intramuscular (IM) injection in the hind thighs. Prime and booster doses of vaccine were given at 21 days intervals. When used, 7HP349 (25 µg/50 μL vehicle) was delivered by intraperitoneal injection once daily for five days starting one day following vaccination. For prophylactic studies, mice were immunized on day 0 and day 21, challenged on day 42, and euthanized on day 63 corresponding to acute parasitemic phase^53^. For therapeutic studies, mice were infected on day 0, immunized on day 21 and day 42, and euthanized on day 110 corresponding to the chronic phase^54^. Age-matched, non-vaccinated/non-infected, and non-vaccinated/infected mice were used as negative and positive controls, respectively. The schematic view of immunization and challenge timeline is presented in Fig. 2c, d.

### Flow cytometry characterization of T cells

Single-cell suspensions of spleen from mice (*n* ≥ 5 mice per group) were prepared by standard methods and splenocytes were incubated with red blood cell lysis buffer (Sigma) and washed with cold 1× PBS. For ex vivo profile of T cells, splenocytes were suspended in brilliant stain buffer and incubated for 10 min at 4 °C with anti-mouse CD16/CD32 Fc block (BD Biosciences, San Jose, CA). Cells were washed twice, resuspended in 50 µL of flow cytometry staining buffer (00-4222-26, eBioscience, San Diego, CA), and labeled with a cocktail of fluorochrome-conjugated cell surface antibodies (concentration determined by titration) for 30 min at 4 °C in dark. Cells were washed, fixed, and permeabilized with cytofix/cytoperm solution for 20 min, washed with perm wash buffer (BD Biosciences, San Jose, CA), and utilized for intracellular staining of markers of functional activation. Tubes containing unstained cells and cells incubated with isotype-matched IgG, FMO (fluorescence minus one), and live/dead stain were included as controls^38^. All antibodies used for flow cytometry are listed in Supplementary Table 4.

For studying the splenocytes’ recall response, *T. cruzi* trypomastigotes (1 × 10^9^ trypomastigotes/mL PBS) were lysed by repeated freeze-thaw method, and soluble *Tc* trypomastigotes antigenic extract was used for in vitro stimulation^55^. Splenocytes were suspended in color-free RPMI media (4 × 10^5^ cells/100 μL), and incubated with or without *Tc* trypomastigotes antigenic lysate (25 μg/mL) at 37 °C, 5% CO_2_ for 48 h. For the measurement of intracellular cytokines and immune cell activation markers, brefeldin A (10 μg/mL, BD Biosciences) was added to splenocyte cultures for the final 4 h of incubation to block protein secretion. Staining of surface and intracellular molecules were performed as above.

All samples were visualized on an LSRII Fortessa Cell Analyzer (BD Biosciences), acquiring 1–2 × 10^6^ live events. Total lymphocytes from each group were gated for CD3^+^T cells after removing doublets, dead cells, and applying CD3^+^ FMO control (Supplementary Fig. 1a–i). The concatenated CD3^+^ populations from all the categorial treatments were downsampled to 1 × 10^5^ cells. We applied FlowSOM FlowJo plugin with grid dimensions 7 × 7. The resultant grid created 49 nodes which were allowed to distribute into unbiased self-organizing 14 metaclusters named P0-P13 (Supplementary Fig. 1s). The concatenated fcs files for each group were submitted to t-Distributed Stochastic Neighbor Embedding (t-SNE) analysis (Supplementary Fig. 1k–r) that allows reduction of dimensionality and is well suited for the visualization of high-dimensional datasets using parameters: iteration = 1000, perplexity = 30, and learning rate (eta) = 4900^56^. We used median fluorescence intensity (MFI) of CD4, CD8, CD25, CD62L, and CD44 on the CD3 + lymphocytes for identification of T cell sub-populations in the metaclusters (Supplementary Fig. 1t). The MFI of intercellular IFN-γ (Th1 cytokine) and cytolytic molecules (PFN and GZB) on specific T cell subpopulations were also captured by flow cytometry.

### Cytokine’s release

Splenocytes of non-vaccinated/non-infected, non-vaccinated/infected, and vaccinated/infected mice were stimulated in vitro with *Tc* trypomastigotes antigenic lysate as above, and culture supernatants were used for the measurement of TNF-α, IFN-γ, IL-1β, and IL-10 cytokines by using ELISA kits (Invitrogen)^57^.

### Tissue parasite burden

Heart and skeletal muscles tissues (10 mg) of mice (*n* = 7–12 mice per group) were homogenized, treated with proteinase K, and total DNA was purified by phenol/chloroform extraction and ethanol precipitation. A real-time quantitative PCR was performed on an iCycler thermal cycler with SYBR Green Supermix (Bio-Rad), 50 ng of total DNA, and oligonucleotides specific for *Tc18SrDNA* (forward, 5′-TTTTGGGCAACAGCAGGTCT-3′; reverse, 5′-CTGCGCCTACGAGACATTCC-3′; amplicon size: 199 bp) and murine *GAPDH* (forward, 5′-AACTTTGGCATTGTGGAAGG-3′; reverse, 5′-ACACATTGGGGGTAGGAACA-3′; amplicon size: 223 bp). Each sample was analyzed in duplicate, and the threshold cycle (*C*_*T*_) values for *Tc*18SrDNA were normalized to Ct values of *GAPDH* reference cDNA. The relative parasite burden (i.e., *Tc18SrDNA* level) was calculated by 2^−ΔCt^ method, where Δ*Ct* represents the C_t_ (target) -C_t_ (reference)^58^.

### Histology

Tissue sections of heart and skeletal muscle from mice were fixed in 10% buffered formalin for 24 h, dehydrated in absolute alcohol, cleared in xylene, and embedded in paraffin. Paraffin-embedded 5-micron tissue sections were stained with hematoxylin and eosin (H&E) and Masson’s Trichrome to examine inflammatory infiltrate and collagen deposition, respectively. Heart and skeletal muscle tissue slides (n ≥3 mice per group, at least two slides per tissue per mouse, nine microscopic fields per slide) were analyzed by light microscopy (20X magnification). H&E stained tissue sections were scored for inflammation as (0)-absent/none, (1)-focal or mild with ≤1 foci, (2)-moderate with ≥2 inflammatory foci, (3)-extensive with generalized coalescing of inflammatory foci or disseminated inflammation, and (4)-severe with diffused inflammation, interstitial edema, and loss of tissue integrity^59,60^. Fibrosis was assessed by measuring the Masson’s Trichrome-stained collagen area as a percentage of the total myocardial area using Simple PCI software (version 6.0; Compix, Sewickley, PA) connected to an Olympus polarizing microscope system (Center Valley, PA). All pixels with blue stain in Masson’s trichrome-stained sections were selected to build a binary image, subsequently calculating the total area occupied by connective tissue. Sections were categorized based on % fibrotic area: (0) <1%, (1) 1–5%, (2) 5–10%, (3) 10–15%, and (4) >15%^59,60^.

### Statistical analysis

Data were analyzed by using an SPSS (version 14.0, SPSS Inc, Chicago, Illinois) or GraphPad InStat ver.5 software. D’Agostino and Pearson omnibus normality test was performed to check the normal distribution of data. Significance (*p* values) between control vs. infected groups was calculated by unpaired *t*-test or Mann–Whitney U test. *P* values comparing multiple groups were calculated by 1-way analysis of variance (ANOVA) with Tukey’s post-hoc test. Significance is annotated as described in figure legends.

### Reporting summary

Further information on research design is available in the Nature Research Reporting Summary linked to this article.

## Supplementary information

Supplementary Information

Reporting Summary

## Data Availability

All data are provided within the manuscript.
