# Supplementary material for: Use of a small molecule integrin activator as a systemically administered vaccine adjuvant in controlling Chagas disease
Source: NPJ Vaccines. 2021 Sep 8;6:114. doi: 10.1038/s41541-021-00378-5 (PMC8426359; doi:10.1038/s41541-021-00378-5)
Supplement: Supplementary file 1 — Supplementary Information [file 41541_2021_378_MOESM1_ESM.pdf]

Supplementary Material

Use of a small molecule integrin activator as a systemically administered vaccine adjuvant in controlling Chagas disease

Authors: Nandadeva Lokugamage PhD [1, †], Imran H. Chowdhury PhD [1, †], Ronald J. Biediger PhD [2], Robert V. Market [2], Sayadeth Khounlo [2], Navin D. Warier [2], Shen-An Hwang PhD [3], Jeffrey K. Actor PhD [3], Darren G. Woodside PhD [2], Upendra Marathi PhD [4], Peter Vanderslice PhD [2, *], Nisha Jain Garg PhD [1, 5, *]

Affiliations:

[1] Department of Microbiology and Immunology, University of Texas Medical Branch (UTMB), Galveston, TX, USA

[2] Department of Molecular Cardiology, Texas Heart Institute, Houston, TX

[3] Department of Pathology and Laboratory Medicine, UTHealth McGovern Medical School, Houston, TX

[4] 7 Hills Pharma LLC, 2450 Holcombe Blvd, Suite J, Houston TX

[5] Institute for Human Infections and Immunity, UTMB, Galveston TX

***** Corresponding authors: Dr. Nisha Jain Garg, O: 409-747-6865; E: nigarg@utmb.edu, Dr. Peter Vanderslice, O: 832-355-9083, E: pvanderslice@texasheart.org

† Co-first authors, contributed equally

Figure S1

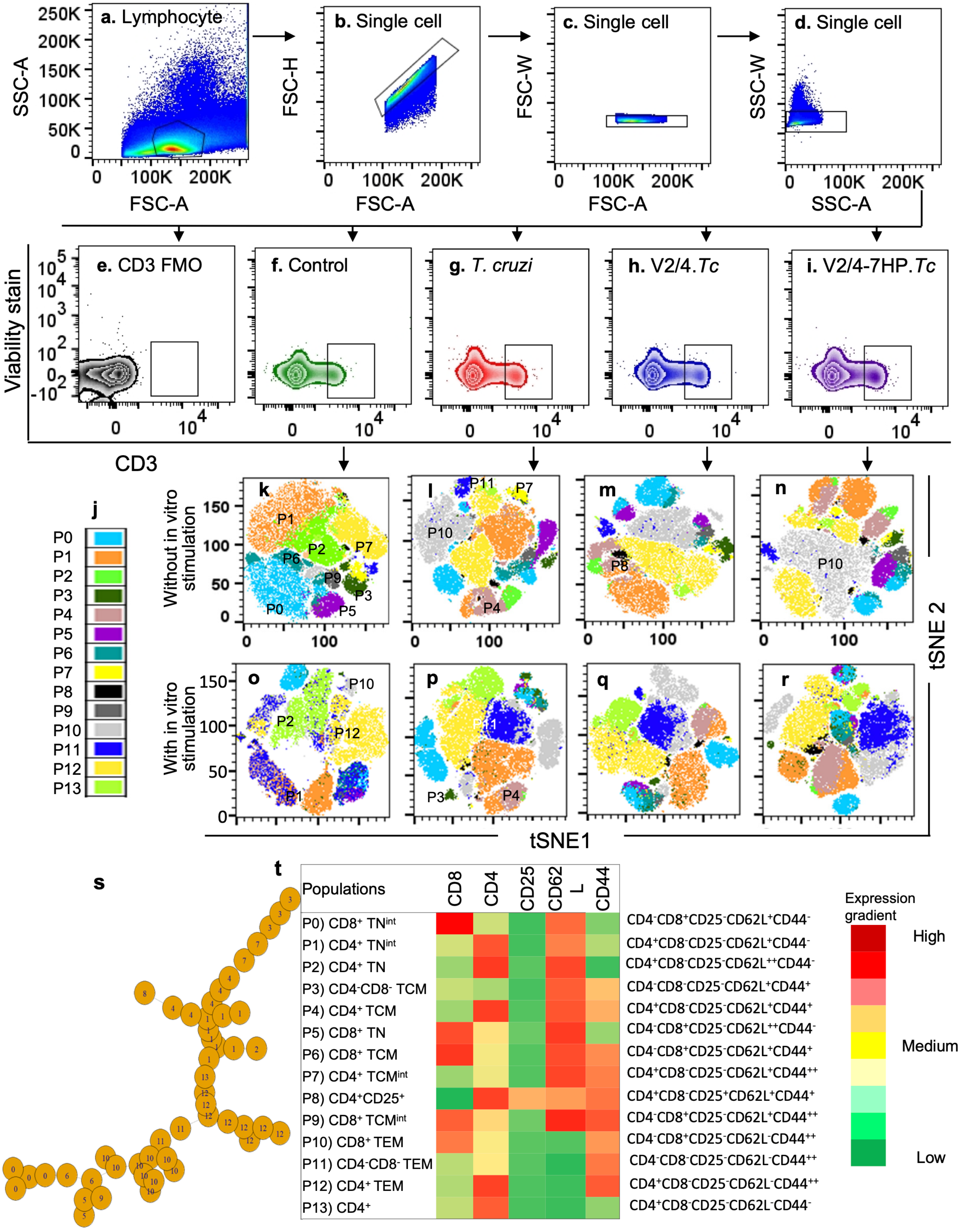

a. Lymphocyte
SSC-A
FSC-A
b. Single cell
FSC-H
FSC-A
c. Single cell
FSC-W
FSC-A
d. Single cell
SSC-W
SSC-A
e. CD3 FMO
f. Control
g. *T. cruzi*
h. V2/4.*Tc*
i. V2/4-7HP.*Tc*
Viability stain
CD3
j
P0
P1
P2
P3
P4
P5
P6
P7
P8
P9
P10
P11
P12
P13
Without in vitro stimulation
With in vitro stimulation
k
l
m
n
o
p
q
r
tSNE 2
tSNE1
s
t
Populations
CD8
CD4
CD25
CD62L
CD44
P0) CD8$^+$ TN^{int}
P1) CD4$^+$ TN^{int}
P2) CD4$^+$ TN
P3) CD4$^-$CD8$^-$ TCM
P4) CD4$^+$ TCM
P5) CD8$^+$ TN
P6) CD8$^+$ TCM
P7) CD4$^+$ TCM^{int}
P8) CD4$^+$CD25$^+$
P9) CD8$^+$ TCM^{int}
P10) CD8$^+$ TEM
P11) CD4$^-$CD8$^-$ TEM
P12) CD4$^+$ TEM
P13) CD4$^+$
CD4$^-$CD8$^+$CD25$^-$CD62L$^+$CD44$^-$
CD4$^+$CD8$^-$CD25$^-$CD62L$^+$CD44$^-$
CD4$^+$CD8$^-$CD25$^-$CD62L^{++}CD44$^-$
CD4$^-$CD8$^-$CD25$^-$CD62L$^+$CD44$^+$
CD4$^+$CD8$^-$CD25$^-$CD62L$^+$CD44$^+$
CD4$^-$CD8$^+$CD25$^-$CD62L^{++}CD44$^-$
CD4$^-$CD8$^+$CD25$^-$CD62L$^+$CD44$^+$
CD4$^+$CD8$^-$CD25$^-$CD62L$^+$CD44^{++}
CD4$^+$CD8$^-$CD25$^+$CD62L$^+$CD44$^+$
CD4$^-$CD8$^+$CD25$^-$CD62L$^+$CD44^{++}
CD4$^-$CD8$^+$CD25$^-$CD62L$^-$CD44^{++}
CD4$^-$CD8$^-$CD25$^-$CD62L$^-$CD44^{++}
CD4$^+$CD8$^-$CD25$^-$CD62L$^-$CD44^{++}
CD4$^+$CD8$^-$CD25$^-$CD62L$^-$CD44$^-$
Expression gradient
High
Medium
Low

Supplementary Figure 1. Representative gating strategy for capturing of CD3^{+} T cell subpopulations followed by analysis. C57BL/6 female mice were immunized, challenged, and euthanized as in **Fig.2c**. Splenocytes were stained with 10-color fluorochrome. **(a)** Lymphocytes were captured from acquired cells in LSRII Fortessa. After removing doublets **(b-d)**, CD3^{+} FMO control **(e)** was used to gate CD3^{+} live cells from control **(f)**, infected **(g)**, V2/4.*Tc* **(h)** and V2/4-7HP349.*Tc* **(i)** groups of mice. FlowSOM analysis was performed on CD3^{+} live cells from all the groups to generate unbiased tree of self-organizing meta-clusters (named P0-P13), shown in **j**. The tSNE (t-distributed stochastic neighbor embedding) plots were generated with non-linear reduction method to visualize the 14 sub-populations in two-dimensional space in control (non-vaccinated/non-infected) **(k)**, non-vaccinated/infected **(l)**, V2/4.*Tc* **(m)** and V2/4-7HP349.*Tc* **(n)** groups of mice. Another set of FlowSOM analysis was performed to show tSNE plots after in vitro stimulation with *Tc* antigenic lysates of splenocytes from control **(o)**, infected **(p)**, V2/4.*Tc* **(q)** and V2/4-7HP349.*Tc* **(r)** groups of mice. **(s)** Unbiased tree of self-organizing meta-clusters (named P0-P13) of CD3^{+}T cells based on cell surface expression of CD4, CD8, CD25, CD62L, and CD44 markers. **(t)** Relative median fluorescent intensities of surface receptors from all the cohorts were utilized to visualize as heat-map for 14 meta-clusters of CD3^{+} live T cells. Each row represents a sub-population based on expression of markers presented in the columns. The percentages of cells expressing surface markers were visualized with color scale from green (0) to red (1000), where green and red show decrease and increase in expression, respectively. Metaclusters were analyzed for median fluorescence intensity of IFN-gamma, Perforin and Granzyme B. Gating panel(s) corresponding to data panel(s) in figure 3 and figure 4 of the manuscript are as following:

Metaclusters	Populations	Supplementary Fig.1 panels - No in vitro stimulation	Supplementary Fig.1 - with in vitro stimulation	Corresponding panels in the manuscript figures
P11	CD4^{-}CD8^{-} TEM	k, l, m, n	o, p, q, r	3a
P3	CD4^{-}CD8^{-} TCM	k, l, m, n	o, p, q, r	3b, 4a, 4f and 4k
P2	CD4^{+} TN	k, l, m, n	o, p, q, r	3c
P1	CD4^{+} TN^{int}	k, l, m, n	o, p, q, r	3d
P12	CD4^{+} TEM	k, l, m, n	o, p, q, r	3e, 4b, 4g and 4l
P7	CD4^{+} TCM^{int}	k, l, m, n	o, p, q, r	3f
P4	CD4^{+} TCM	k, l, m, n	o, p, q, r	3g, 4c, 4h and 4m
P5	CD8^{+} TN	k, l, m, n	o, p, q, r	3h
P0	CD8^{+} TN^{int}	k, l, m, n	o, p, q, r	3i
P10	CD8^{+} TEM	k, l, m, n	o, p, q, r	3j, 4d, 4i and 4n
P9	CD8^{+} TCM^{int}	k, l, m, n	o, p, q, r	3k
P6	CD8^{+} TCM	k, l, m, n	o, p, q, r	3l, 4e, 4j and 4o

Abbreviations: TEM: T effector/effector memory, TCM: T central memory, T^{int}: T intermediate, TN, T naïve.

Supplementary Table 1. Splenic T cell profile in *T. cruzi* infected mice (± vaccine).

Pop#	Population name	No in vitro stimulation								With in vitro stimulation with *Tc* antigenic lysate					
		Control		*T. cruzi*		V2/4.*Tc*		V2/4-7HP.*Tc*		*T. cruzi*		V2/4.*Tc*		V2/4-7HP.*Tc*	
		Mean	±SEM	Mean	±SEM	Mean	±SEM	Mean	±SEM	Mean	±SEM	Mean	±SEM	Mean	±SEM
11	CD4$^-$CD8$^-$ TEM	1.11	0.18	2.19	0.58**	2.19	0.31	1.73	0.17	19.38	1.71	17.13	2.73	18.80	1.16
3	CD4$^-$CD8$^-$ TCM	3.79	0.89	1.81	0.43**	2.79	1.43	1.83	0.55	4.67	1.50	1.45	0.29	7.50	3.29$^{\&/\wedge\wedge}$
13	CD4$^+$ premature	0.80	0.25	1.08	0.23	1.27	0.39	1.19	0.33	1.19	0.40	1.24	0.47	2.39	0.46
2	CD4$^+$ TN	14.41	3.54	2.82	0.99***	1.79	0.66	1.74	0.26	12.56	3.88	10.75	5.07	3.76	2.11$^{\&/\wedge}$
1	CD4$^+$ TN^{int}	28.38	5.06	22.64	8.34	16.94	8.50	12.70	4.14$^{\&}$	14.21	2.46	12.33	3.33	7.22	1.40$^{\&/\wedge}$
12	CD4$^+$ TEM	13.20	0.92	20.44	0.97***	29.02	2.14$^{\#\#\#}$	20.11	1.64^^^	11.39	2.16	12.03	3.71	17.74	1.50$^{\&/\wedge}$
7	CD4$^+$ TCM^{int}	1.84	0.57	1.87	0.51	1.19	0.12	1.51	0.13	0.34	0.10	2.53	2.13	0.47	0.09
4	CD4$^+$ TCM	0.47	0.27	6.86	1.29***	5.79	1.60	7.79	1.16	4.56	1.34	5.23	1.89	10.62	1.50$^{\&/\wedge}$
5	CD8$^+$ TN	4.51	2.79	4.81	1.60	3.81	1.50	4.54	0.92	0.88	0.21	1.14	0.39	1.60	0.29
0	CD8$^+$ TN^{int}	20.57	3.53	9.74	2.87**	8.72	3.75	5.60	1.51$^{\&}$	6.33	1.79	4.45	1.40	5.12	1.00
10	CD8$^+$ TEM	2.23	0.22	20.53	6.83***	21.48	7.33	36.00	6.03$^{\&\&\&/\wedge\wedge\wedge}$	13.80	2.15	17.86	2.33$^{\#}$	15.50	2.35^
9	CD8$^+$ TCM^{int}	2.20	1.36	1.63	0.57	1.52	0.62	2.09	0.34	5.54	1.74	6.25	3.11	5.37	2.94
6	CD8$^+$ TCM	6.07	0.71	2.90	0.33***	2.30	0.35	2.06	0.10	1.01	0.29	1.83	0.67	2.39	0.43
8	CD4$^+$CD25$^+$	0.43	0.20	0.69	0.18	1.16	0.43	1.09	0.41	4.14	1.35	5.80	2.79	1.48	0.31$^{\&/\wedge}$

C57BL/6 female mice were prophylactically immunized with V2/4 in presence or absence of 7H349, challenged with *T. cruzi,* and euthanized at 21 days post-infection. Splenocytes were either analyzed immediately or in vitro stimulated with *T. cruzi* antigenic lysate for 48 h. Cells were labeled with flurochrome-conjugated antibodies and analyzed by flow cytometry. Self-organizing meta-clusters of CD3$^+$T cells based on the expression levels of CD4, CD8, CD25, CD62L, and CD44 antigens were formed by FlowSOM analysis. The frequencies (percentage of total T cells) of the 14 metaclusters (labeled as P0-P13) in mice are presented.

Data are plotted as mean values ± SEM (n=5-10 mice per group). Significance between control vs. infected groups (*) was calculated by unpaired t test or Mann-Whitney U test. Significance among infected groups was calculated by 1-way analysis of variance (ANOVA) with Tukey's post-hoc test (comparison of multiple groups) and plotted as $^{\#}$infected vs. V2/4.*Tc*, ^infected vs. V2/4-7HP349.*Tc*, and $^{\&}$V2/4-7HP349 vs. V2/4. P values of <0.05, <0.01, and <0.001 are annotated with one, two, and three symbols, respectively.
Abbreviations: TEM: T effector/effector memory, TCM: T central memory, T^{int}: T intermediate, TN, T naïve.

Supplementary Table 2. Median fluorescence intensity (MFI) of functional markers of splenic T cell activation in infected mice prophylactically treated with candidate vaccine (± 7HP349).

Pop#	Population name	No in vitro stimulation								With in vitro stimulation with *Tc* antigenic lysate							
		Control		*T. cruzi*		V2/4.*Tc*		V2/4-7HP.*Tc*		Control		*T. cruzi*		V2/4.*Tc*		V2/4-7HP.*Tc*	
		Mean	±SEM	Mean	±SEM	Mean	±SEM	Mean	±SEM	Mean	±SEM	Mean	±SEM	Mean	±SEM	Mean	±SEM
Interferon gamma (MFI)																	
3	DN TCM	-137.8	14.31	-141.6	22.07	-130.8	23.61	-116.0	8.44	67.67	155.73	-94.95	103.51	-118.4	60.99	-32.38	30.5
12	CD4$^+$ TEM	14.8	4.19	89.77	28.27*	97.34	39.91	106.5	19.25	52.55	11.43	110.46	33.26***	170.64	38.18##	151.21	23.76&&
4	CD4$^+$ TCM	33.84	4.36	107.87	26.91*	122.42	38.94	141.68	17.15&	50.34	139.38	88.03	77.51**	274.63	34.32#	288.11	12.58&
10	CD8$^+$ TEM	21.89	3.83	56.67	8.37***	59.64	13.98	73.74	16.4	66.73	19.94	121.85	7.19**	169.59	17.61##	225.96	4.28&&&/^^
6	CD8$^+$ TCM	2.9	0.65	5.46	0.97*	199.82	28.19###	223.2	7.64&&&	72.36	22.11	131.4	8.78***	160.11	24.23	203.44	16.12&
Perforin (MFI)																	
3	DN TCM	42.88	7.95	114.67	6.35***	148.44	6.84	183.34	12.89&&&/^^	120.66	14.67	151.21	31.43**	203.89	37.79#	248.98	27.67&&
12	CD4$^+$ TEM	51.6	8.83	98.71	11.1**	154.92	12.82##	184.5	10.03&&&/^	49.4	7.48	129.76	18.05***	173.3	22.53	182.23	17.12&&
4	CD4$^+$ TCM	58.55	7.21	140.86	7.03***	148.4	13.01	148.19	6.98	114.11	18.97	162.89	22.41**	217.6	30.29	243.61	22.58&
10	CD8$^+$ TEM	30.85	5.93	124.86	10.56***	145.42	11.73	178.13	12.27&&	116.75	15.45	166.5	13.02**	206.74	17.89	274.89	15.47&&&
6	CD8$^+$ TCM	60.44	9.9	148	11.44***	159.34	12.88	199.63	12.44&/^	76.87	3.88	172.06	34.18***	250.25	34.13	287.44	23.38&&
Granzyme (MFI)																	
3	DN TCM	57.24	11.41	58.69	3.56	56.1	3.12	63.16	2.25	55.06	12.52	54.26	12.58	70.49	17.72	94.32	16.55&
12	CD4$^+$ TEM	75.84	5.01	78.33	4.61	85.28	8.68	86.18	2.69	54.68	16.49	95.19	3.97	112.1	11.36	115.07	15.42
4	CD4$^+$ TCM	50.48	5.2	55.63	5.33	58.02	2.27	58.83	2.97	47.49	10.43	48.97	10.81	74.18	10.14	106.11	3.51&&&
10	CD8$^+$ TEM	72.58	4.75	89.09	7.13*	149.3	7.42###	153.66	5.07&&&	61.18	14.03	92.43	6.57	177.11	12.43##	208.91	5.5&&&
6	CD8$^+$ TCM	41.91	3.18	69.19	6.94**	68.88	2.21	70.88	3.07	58.6	13.94	80.1	11.46**	81.75	16.84	101.41	15.45

C57BL/6 female mice were prophylactically immunized with V2/4 in presence or absence of 7H349, challenged with *T. cruzi,* and euthanized at 21 days post-infection. Splenocytes were either analyzed immediately or in vitro stimulated with *T. cruzi* antigenic lysate Ag Lys) for 48 h. Cells were labeled with fluorochrome-conjugated antibodies and analyzed by flow cytometry. First, self-organizing meta-clusters of CD3$^+$T cells based on the expression levels of CD4, CD8, CD25, CD62L, and CD44 antigens were formed by FlowSOM analysis. The median fluorescent intensities of IFN-γ, perforin (PFN) and granzyme B (GZB) in double negative (DN), CD4$^+$, and CD8$^+$ TEM and TCM metaclusters was examined.

Data are plotted as mean values ± SEM (n=5-10 mice per group). Significance between control vs. infected groups (*) was calculated by unpaired t test or Mann-Whitney U test. Significance among infected groups was calculated by 1-way analysis of variance (ANOVA) with Tukey's post-hoc test (comparison of multiple groups) and plotted as #infected vs. V2/4.*Tc*, ^infected vs. V2/4-7HP349.*Tc*, and &V2/4-7HP349 vs. V2/4. P values of <0.05, <0.01, and <0.001 are annotated with one, two, and three symbols, respectively. Abbreviations: TN, Naïve T cells; TCM, central memory T cells; TEM, effector/effector memory T cells; int, intermediate.

Supplementary Table 3: Semiquantitative scoring of tissues sections for inflammation and fibrosis in infected mice treated with candidate vaccine (± 7HP349).

Groups	Heart	Skeletal muscle
Prophylactic efficacy: inflammation score		
None	0	0
T. cruzi	2-3 (2.69 ± 0.47)***	3-4 (3.51 ± 0.52)***
V2/4.*Tc*	0-1 (0.55 ± 0.51)$^{***,\#\#\#}$	1-2 (1.83 ± 0.38)$^{***,\#\#\#}$
V2/4-7HP.*Tc*	0-1 (0.72 ± 0.46)$^{***,\&\&\&}$	1-2 (1.17 ± 0.38)$^{***,\&\&\&,\wedge\wedge\wedge}$
Therapeutic efficacy: inflammation score		
None	0	0
T. cruzi	0-3 (2.17 ± 0.17)***	1-3 (2.92 ± 0.08)***
V2/4.*Tc*	0-1 (0.82 ± 0.11)$^{***,\#\#\#}$	1 (1.0 ± 0)$^{***,\#\#\#}$
V2/4-7HP.*Tc*	0-1 (0.58 ± 0.14)$^{***,\&\&\&}$	0-1 (0.42 ± 0.14)$^{***,\&\&\&,\wedge\wedge}$
Therapeutic efficacy: fibrosis score		
None	0	0
T. cruzi	5.1 ± 0.48***	6.4 ± 0.08***
V2/4.*Tc*	0.73 ± 0.02$^{***,\#\#}$	0.77± 0.05$^{***,\#\#}$
V2/4-7HP.*Tc*	0.33 ± 0.06$^{***,\&\&,\wedge}$	0.33 ± 0.05$^{***,\&\&,\wedge}$

C57BL/6 female mice were prophylactically immunized with V2/4 in presence or absence of 7H349, challenged with *T. cruzi,* and euthanized at 21 days post-infection. Paraffin-embedded 5 µm heart and skeletal muscle tissue sections were examined by hematoxylin/eosin (H&E) staining (magnification: 20X). The inflammatory score and fibrosis score are presented as mean values ± SEM, derived from n=3 mice per group, 2 tissue sections per mouse, >9 microscopic fields per tissue. Significance between control vs. infected (*) was calculated by unpaired t test or Mann-Whitney U test. Significance among infected groups was calculated by 1-way analysis of variance (ANOVA) with Tukey's post-hoc test (comparison of multiple groups) and plotted as $^{\#}$infected vs. V2/4.*Tc*, ^infected vs. V2/4-7HP349.*Tc*, and $^{\&}$V2/4-7HP349 vs. V2/4. P values of <0.05, <0.01, and <0.001 are annotated with one, two, and three symbols, respectively.

Supplementary Table 4. Antibodies used for flow cytometry analysis of T cells

Marker	Color	Ex/ Em (nm)	Antibody cat #	Source
CD3 epsilon	APC	640/670±15	Hamster α m / IgG1, κ (145-2C11)	BD Biosciences (553066)
CD4	BV510	405 / 525±25	Rat α m / IgG2a, κ (RM4-5)	BD Biosciences (563106)
CD8	BUV395	355/450±25	Rat α m / IgG2a, κ (53-6.7)	BD Biosciences (563786)
Granzyme B	PE	561/582±7.5	Rat α m / IgG2a, κ (NGZB)	eBiosciences (12-8898-82)
IFN-γ	BV711	405/710±25	Rat α m / IgG1, κ (XMG1.2)	BD Biosciences (564336)
Perforin	FITC	488/530±15	Rat α m / IgG2a, κ (eBioOMAK-D)	Thermo Fisher (11-9392-82)
CD62L	BV650	405/670±15	Rat α m/ IgG2a, κ (MEL-14)	BD Biosciences (564108)
CD44	BV786	405/780±30	Rat α m / IgG2b, κ (IM7)	BD Biosciences (563736)
CD25	BV421	405/450±25	Rat α m / IgM, κ (7D4)	BD Biosciences (564571)
Fixable viability stain		759/780		BD Biosciences (565388)
