# Supplementary material for: Use of a small molecule integrin activator as a systemically administered vaccine adjuvant in controlling Chagas disease
Source: NPJ Vaccines. 2021 Sep 8;6:114. doi: 10.1038/s41541-021-00378-5 (PMC8426359; doi:10.1038/s41541-021-00378-5)
Supplement: Supplementary file 2 — Reporting Summary [file 41541_2021_378_MOESM2_ESM.pdf]

nature research

Corresponding author(s): Nisha J Garg; Peter Vanderslice

Last updated by author(s): 03/29/2021

Reporting Summary

Nature Research wishes to improve the reproducibility of the work that we publish. This form provides structure for consistency and transparency in reporting. For further information on Nature Research policies, see our Editorial Policies and the Editorial Policy Checklist.

Statistics

For all statistical analyses, confirm that the following items are present in the figure legend, table legend, main text, or Methods section.

n/a	Confirmed	
☐	☒	The exact sample size (*n*) for each experimental group/condition, given as a discrete number and unit of measurement
☐	☒	A statement on whether measurements were taken from distinct samples or whether the same sample was measured repeatedly
☐	☒	The statistical test(s) used AND whether they are one- or two-sided<br>*Only common tests should be described solely by name; describe more complex techniques in the Methods section.*
☒	☐	A description of all covariates tested
☐	☒	A description of any assumptions or corrections, such as tests of normality and adjustment for multiple comparisons
☐	☒	A full description of the statistical parameters including central tendency (e.g. means) or other basic estimates (e.g. regression coefficient) AND variation (e.g. standard deviation) or associated estimates of uncertainty (e.g. confidence intervals)
☒	☐	For null hypothesis testing, the test statistic (e.g. *F*, *t*, *r*) with confidence intervals, effect sizes, degrees of freedom and *P* value noted<br>*Give P values as exact values whenever suitable.*
☒	☐	For Bayesian analysis, information on the choice of priors and Markov chain Monte Carlo settings
☒	☐	For hierarchical and complex designs, identification of the appropriate level for tests and full reporting of outcomes
☒	☐	Estimates of effect sizes (e.g. Cohen's *d*, Pearson's *r*), indicating how they were calculated

Our web collection on statistics for biologists contains articles on many of the points above.

Software and code

Policy information about availability of computer code

Data collection	N/A. Routine tools, e.g., excel were used for data collection
Data analysis	N/A. Routine software, e.g., GraphPad Prism were used for data analysis.

For manuscripts utilizing custom algorithms or software that are central to the research but not yet described in published literature, software must be made available to editors and reviewers. We strongly encourage code deposition in a community repository (e.g. GitHub). See the Nature Research guidelines for submitting code & software for further information.

Data

Policy information about availability of data

All manuscripts must include a data availability statement. This statement should provide the following information, where applicable:

- Accession codes, unique identifiers, or web links for publicly available datasets
- A list of figures that have associated raw data
- A description of any restrictions on data availability

All data are presented in the manuscript. There is no restriction on data availability.

Field-specific reporting

Please select the one below that is the best fit for your research. If you are not sure, read the appropriate sections before making your selection.

☒ Life sciences ☐ Behavioural & social sciences ☐ Ecological, evolutionary & environmental sciences

For a reference copy of the document with all sections, see nature.com/documents/nr-reporting-summary-flat.pdf

Life sciences study design

All studies must disclose on these points even when the disclosure is negative.

Sample size	Using the data from previous published studies to calculate sample size by nQuery
Data exclusions	None
Replication	2-3 analysis per sample, 2-3 samples per experiment (in vitro) and n>5 in vivo. mean value +/- SEM are derived from all samples included in the study.
Randomization	Mice were randomly distributed to different groups.
Blinding	*Describe whether the investigators were blinded to group allocation during data collection and/or analysis. If blinding was not possible, describe why OR explain why blinding was not relevant to your study.*

Reporting for specific materials, systems and methods

We require information from authors about some types of materials, experimental systems and methods used in many studies. Here, indicate whether each material, system or method listed is relevant to your study. If you are not sure if a list item applies to your research, read the appropriate section before selecting a response.

Materials & experimental systems

n/a	Involved in the study	
☐	☒	Antibodies
☐	☒	Eukaryotic cell lines
☒	☐	Palaeontology and archaeology
☐	☒	Animals and other organisms
☒	☐	Human research participants
☒	☐	Clinical data
☒	☐	Dual use research of concern

Methods

n/a	Involved in the study	
☒	☐	ChIP-seq
☐	☒	Flow cytometry
☒	☐	MRI-based neuroimaging

Antibodies

Antibodies used	Antibodies were purchased from ABD Serotec (Raleigh, NC) (anti-a4 mAb HP2/1 [MCA697] and anti-aL mAb 38 [MCA1848GA]). The anti-b2 mAb MEM48 was from abcam [ab657]. The anti-b1 mAb 33B6 was a gift from B. McIntyre (MD Anderson Cancer Center, Houston, TX). All other antibodies and suppliers are listed in the manuscript.
Validation	mAbs were validated by flow cytometry using cell lines that express these surface receptors. We purchased company validated antibodies. Vendor names, catalog numbers are provided along with the antibodies conceived from the companies.

Eukaryotic cell lines

Policy information about cell lines

Cell line source(s)	The cell lines Jurkat, HSB, and 70Z/3 were obtained from American Type Culture Collection (Manassus, VA) and were maintained in recommended culture media. Primary cells isolated from mouse spleen were also used.
Authentication	Cell lines were validated for expression of specific integrins by flow cytometry and adhesion to specific substrates.
Mycoplasma contamination	Not tested.
Commonly misidentified lines (See ICLAC register)	None.

Animals and other organisms

Policy information about studies involving animals; ARRIVE guidelines recommended for reporting animal research

Laboratory animals	6-8 week-old male and female C57BL/6 mice
Wild animals	None
Field-collected samples	None
Ethics oversight	All animal experiments were conducted following the National Institutes of Health guidelines for housing and care of laboratory animals and in accordance with protocols approved by the Institutional Animal Care and Use Committees at The University of Texas Medical Branch at Galveston (protocol number 08-05-029) and the UTHealth McGovern Medical School. All experiments were conducted in ABSL2/BSL2-approved laboratory and all personnel have received appropriate ABSL2/BSL2 training.

Note that full information on the approval of the study protocol must also be provided in the manuscript.

Flow Cytometry

Plots

Confirm that:

☒ The axis labels state the marker and fluorochrome used (e.g. CD4-FITC).

☒ The axis scales are clearly visible. Include numbers along axes only for bottom left plot of group (a 'group' is an analysis of identical markers).

☒ All plots are contour plots with outliers or pseudocolor plots.

☒ A numerical value for number of cells or percentage (with statistics) is provided.

Methodology

Sample preparation	Mentioned in the methodology section of the manuscript
Instrument	LSRII Fortessa Cell Analyzer, BD Biosciences, Serial number: H80000007
Software	FlowSOM plugin in FlowJo software (V10)
Cell population abundance	We did not perform any cell sorting.
Gating strategy	Provided as supplemental figure 1 in the manuscript

☒ Tick this box to confirm that a figure exemplifying the gating strategy is provided in the Supplementary Information.
